# Demonstration of *in vivo* efficacy, cryo-EM-epitope identification, and breadth of two anti-alphavirus bispecific single domain antibodies

**DOI:** 10.1128/jvi.01875-25

**Published:** 2025-12-16

**Authors:** Christina L. Gardner, Sergei Pletnev, Jinny L. Liu, George P. Anderson, Lisa C. Shriver-Lake, Tatsiana Bylund, Courtney Green, Tyler Stephens, Matthew S. Sutton, Yaroslav Tsybovsky, Mario Roederer, Peter D. Kwong, Tongqing Zhou, Ellen R. Goldman, Crystal W. Burke

**Affiliations:** 1 Virology Division, United States Army Medical Research Institute of Infectious Diseases (USAMRIID), Fort Detrick, Frederick, Maryland, USA; 2 Vaccine Research Center, National Institute of Allergy and Infectious Diseases, National Institutes of Health, Bethesda, Maryland, USA; 3 Center for Biomolecular Science and Engineering, U.S. Naval Research Laboratory, Washington, DC, USA; 4 Vaccine Research Center Electron Microscopy Unit, Cancer Research Technology Program, Frederick National Laboratory for Cancer Research, Leidos Biomedical Research, Inc., Frederick, Maryland, USA; 5 Aaron Diamond AIDS Research Center, Vagelos College of Physicians and Surgeons, and Department of Biochemistry and Molecular Biophysics, Columbia University 5798, New York, New York, USA; Loyola University Chicago - Health Sciences Campus, Maywood, Illinois, USA

**Keywords:** alphavirus, Venezuelan equine encephalitis virus, single-domain antibody

## Abstract

Venezuelan equine encephalitis virus (VEEV) is an arbovirus that causes a disease in which 4%–14% of individuals can develop neurological symptoms. Prior to 1970, VEEV was developed as a biological threat agent due to its stability and high morbidity when administered by aerosol. Currently, no FDA-licensed vaccines nor therapeutics for VEEV exist. Single-domain antibodies (sdAbs) may provide a therapeutic option due to their small size and ability to bind recessed epitopes not recognized by conventional antibodies. This study identified two bivalent sdAbs that were able to protect mice from a lethal challenge against both epizootic and enzootic subtypes of VEEV. Cryo-EM structures of sdAb-VEEV complexes revealed the sdAbs that comprised the bivalent sdAbs to recognize a mixture of conserved and non-conserved regions of the VEEV envelope proteins. While all three of the cryo-EM-characterized epitopes were unique in terms of their recognized VEEV residues, two sdAbs, V2B3 and V2C3, overlapped sterically, explaining why only their combinations with the non-sterically overlapping sdAb V3A8f, which composed the bivalent sdAbs described here, were so particularly effective. Binding and neutralization studies found that the bivalent sdAbs have the potential to be broad-spectrum anti-alphavirus therapeutics as they cross-neutralize multiple alphaviruses.

**IMPORTANCE:**

Alphaviruses are no longer geographically constrained to one region of the world but are expanding to be of global concern. In many regions of the world, multiple alphaviruses co-circulate; therefore, having a therapeutic that is pan-alphavirus is important. A cocktail of multiple pan-alphavirus binding/neutralizing antibodies (Abs) may provide optimal coverage against alphaviruses while decreasing the prevalence of viral escape mutants, which could cause the therapeutic to no longer be efficacious. Structures of these Abs, defining their recognition, could assist in identifying optimal combinations. A bivalent pan-alphavirus single-domain antibody could be used in a cocktail with already identified alphavirus IgG antibodies.

## INTRODUCTION

Single-domain antibodies (sdAbs, also called nanobodies or VHHs) are the recombinantly expressed variable domain from camelid heavy chain-only antibodies and provide rugged recognition elements with excellent affinities that can be engineered toward specific applications (1). SdAb properties that are advantageous for therapeutics include good tissue penetration *in vivo*, low immunogenicity, recognition of cryptic epitopes, and ability to tune serum half-life through genetic fusions or PEGylation (2, 3). Additionally, sdAbs have a proven safety profile with the first sdAb-based product, caplacizumab, receiving FDA approval in 2019 (2). Recently, sdAbs have become widely recognized for their potential use as antiviral therapeutics (3, 4), with sdAb-based constructs reported for a range of viruses, including alphaviruses (5, 6), influenza (7–9), coronaviruses (10–12), and filoviruses (13). Constructs in which several sdAbs are linked to form multivalent reagents often provide greater viral neutralization than the un-linked sdAbs (6, 7, 9).

Venezuelan equine encephalitis virus (VEEV) is a mosquito-borne virus that causes periodic epizootic and epidemic outbreaks in equines and humans and has been widely studied because of its potential use as a biothreat agent (14). Although not usually fatal in humans (<1%), VEEV has a high morbidity rate due to the subclinical to clinical ratio being 1:1, can cause neurological disease in 4%–14% of cases, and is fatal in 19%–83% of equine cases (15). All subtypes of VEEV can cause disease in humans. The VEEV IAB and IC subtypes are epizootic strains capable of causing epidemics, while VEEV ID and IE subtypes are enzootic strains mainly transmitted between the mosquito vector and rodent reservoir host. Currently, there are no FDA-approved vaccines or therapeutics for VEEV, and the only treatment after VEEV infection is supportive care. Monoclonal antibodies (mAbs) can be effective in preventing VEEV infection in mice when given therapeutically (16, 17). Additionally, a mAb has been shown to prevent non-human primates from severe disease even when administered 48 h post-exposure (18).

We previously described bivalent sdAb constructs that are able to bind and neutralize VEEV *in vitro* (6). Assayed through plaque reduction and neutralization testing (PRNT), the best constructs reduced 50% of plaques at around 1 ng/mL or lower for both TC-83, the BSL-2 vaccine strain, and the wild-type Trinidad donkey (TrD) strain of the VEEV IAB subtype. Here, we genetically coupled the two-lead bivalent sdAb constructs to an albumin-binding domain and assessed their serum half-life, ability to protect mice from a subcutaneous (SC) and aerosol (AE) VEEV challenge, and ability to protect against multiple subtypes of VEEV. Further, we solved cryo-EM structures to delineate epitope-binding sites, assessed the diversity of the recognized epitopes, and evaluated breadths of binding and neutralization against multiple alphaviruses. Both constructs demonstrated efficacy against multiple subtypes of VEEV and different routes of exposure. Additionally, the sdAbs could bind and/or neutralize multiple different alphaviruses.

## RESULTS

### Single-domain antibody constructs

Previously, bivalent sdAb constructs capable of neutralizing VEEV as determined by PRNT were identified (6). Here, the two most potent neutralizing constructs, CA (composed of sdAb V2C3 linked to sdAb V3A8f) and BA (composed of sdAb V2B3 linked to sdAb V3A8f) were engineered and expressed as genetic fusions with an albumin-binding domain to increase serum half-life (19). The constructs were expressed as fusions with the Alb1 anti-albumin sdAb (20) or the albumin-binding domain (abd) from streptococcal protein G (21) in order to ensure production of a construct that retains potency while integrating a half-life extension property. Both fusion constructs retained the ability to neutralize VEEV as determined by PRNT (Table S1), however, higher yield protein expression was achieved for the bivalent sdAb constructs linked with the abd; therefore, the fusions with the abd were used in follow-on studies to determine the therapeutic potential of the bivalent sdAb constructs (Fig. S1).

### Pharmacokinetics

**Fig 1:**
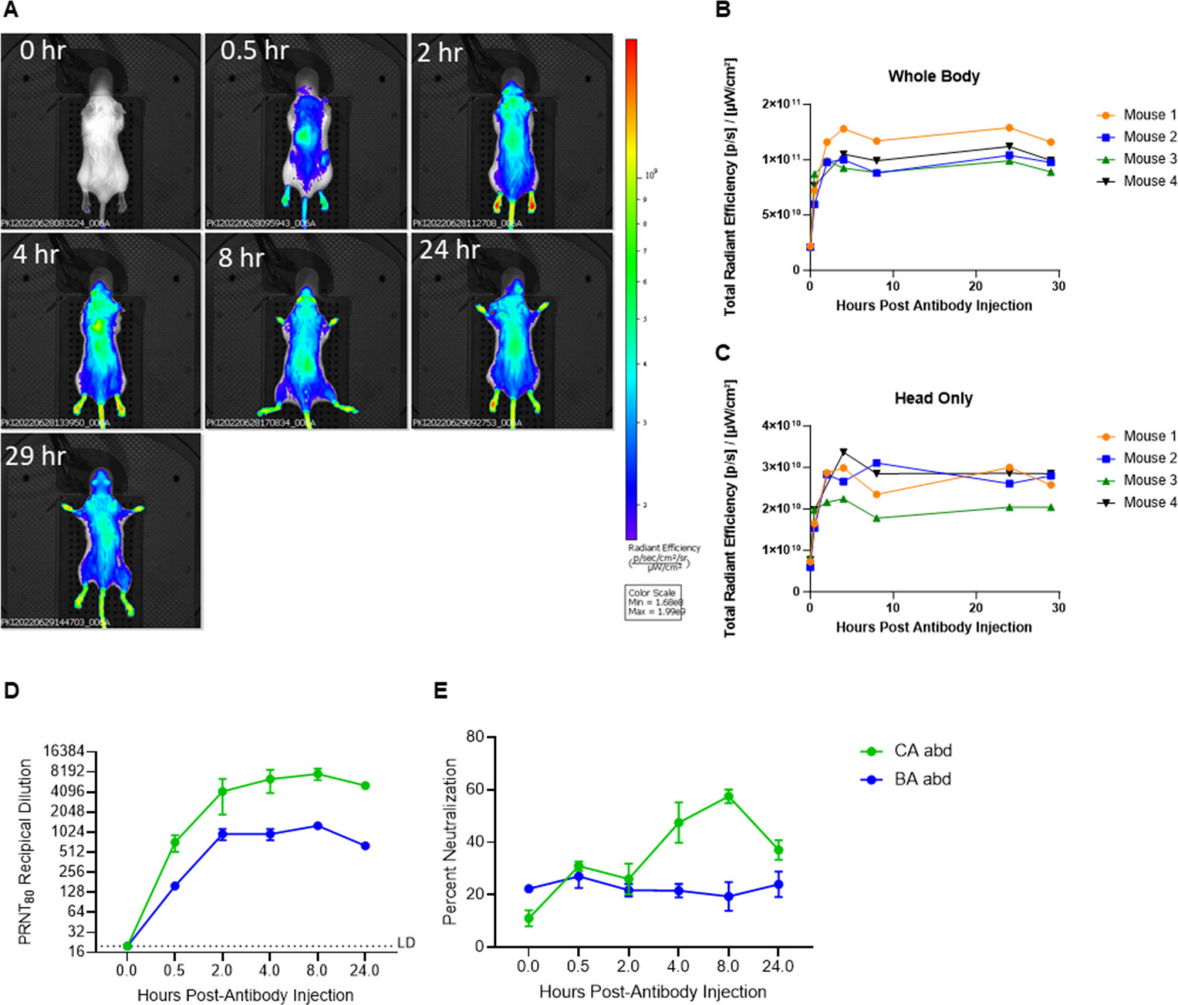
Bioavailability of CA-abd and BA-abd bivalent sdAb. (**A-C**) Imaging of whole mouse injected with dye-labeled PK of CA-abd using *in vivo* imaging system (IVIS). Balb/c mice (*N* = 4) were administered 200 µg of CA-abd, which was labeled with VivoTag 680 XL and imaged using the IVIS spectrum CT. (**A**) IVIS images from a representative mouse from each of the time points. (**B**) Quantification of fluorescent signal from the entire body of the mouse. (**C**) Quantification of fluorescent signal from the head only region of the mouse. (**D-E**) At specific time points post-antibody injection, (**D**) serum and (**E**) brains from mice injected with Ca-abd or BA-abd (*n* = 4/time point) were collected for use in a VEEV PRNT assay. LD = limit of detection.

Prior to evaluating the efficacy of the sdAbs in mice, two pharmacokinetic (PK) studies were performed. First, an imaging PK study using the IVIS Spectrum CT *in vivo* imaging system (IVIS; PerkinElmer) was performed with a fluorescently tagged CA-abd to determine the serum/tissue half-life. The benefits of using the IVIS Spectrum CT were twofold: (i) it reduced the overall number of animals needed to perform the study because (ii) it enabled imaging of the same mouse over time, providing a more accurate estimation of the PK in individual animals. Prior to administering the VivoTag 680 XL-tagged sdAb, mice were imaged to account for any background autofluorescence. After background image collection, four mice received 200 µg of tagged CA-abd through intraperitoneal injection and then were imaged at pre-determined times (0.5, 2, 4, 8, 24, and 29 h) to quantitate the fluorescent signal (Fig. 1A through C). As expected, VivoTag 680 XL-tagged CA-abd was observed disseminated throughout the entire mouse (Fig. 1A). Using the Living Image Software, the fluorescent signal in particular regions of interest (ROIs) was measured. Whole body and head only ROIs (Fig. 1B and C, respectively) were graphed to determine the longevity of CA-abd. These data suggest the half-life of the CA-abd antibody is greater than 29 h.

A potential pitfall with determining serum half-life of the sdAb-abd constructs through *in vivo* imaging is that fluorescently tagging the protein may disrupt the ability of the abd to bind albumin, thereby decreasing the half-life of the sdAb construct. The chemistry used to tag the proteins preferentially targets lysine residues, and the abd contains five lysines. Additionally, a traditional PK study examining the levels of both CA-abd and BA-abd in the serum and the brain at time points up to 24 h post-injection was performed. SdAb constructs were quantified by PRNT showing that the sdAb measured in the serum was functional and able to neutralize the VEEV TrD virus. The serum PK results suggest that both CA-abd and BA-abd possess serum half-lives over 24 h (Fig. 1D), confirming our IVIS PK results. Interestingly, neutralizing antibodies could be detected in brain tissue from animals receiving the CA-abd antibody but not the BA-abd, demonstrating that at least one of the antibodies could reach the central nervous system (Fig. 1E).

### Efficacy against multiple VEEV subtypes

In the VEEV antigenic complex, there are 14 antigenic lineages grouped into six subtypes, with only two lineages that are epizootic strains (IAB and IC), while the rest are enzootic strains (ID-F, II-VI) (22). In just the E1 glycoprotein, there can be up to 29% nucleotide divergence between the six different subtypes of VEEV (23). The FDA guidance for product development under the Animal Rule states that the challenge agent used in efficacy studies should reflect the etiologic agent responsible for the human disease or condition. The VEEV IAB and IC subtypes are epizootic strains of VEEV that have either historically or currently, respectively, caused epidemics. To evaluate the ability of the sdAbs to protect against a wide range of VEEV subtypes, a series of studies were conducted in the BALB/c mouse model of VEEV. Challenges were conducted using a subcutaneous (SC) footpad inoculation to mimic natural infection through mosquito bite or an aerosol exposure (AE) to model a nefarious use of VEEV as a threat agent.

**Fig 2:**
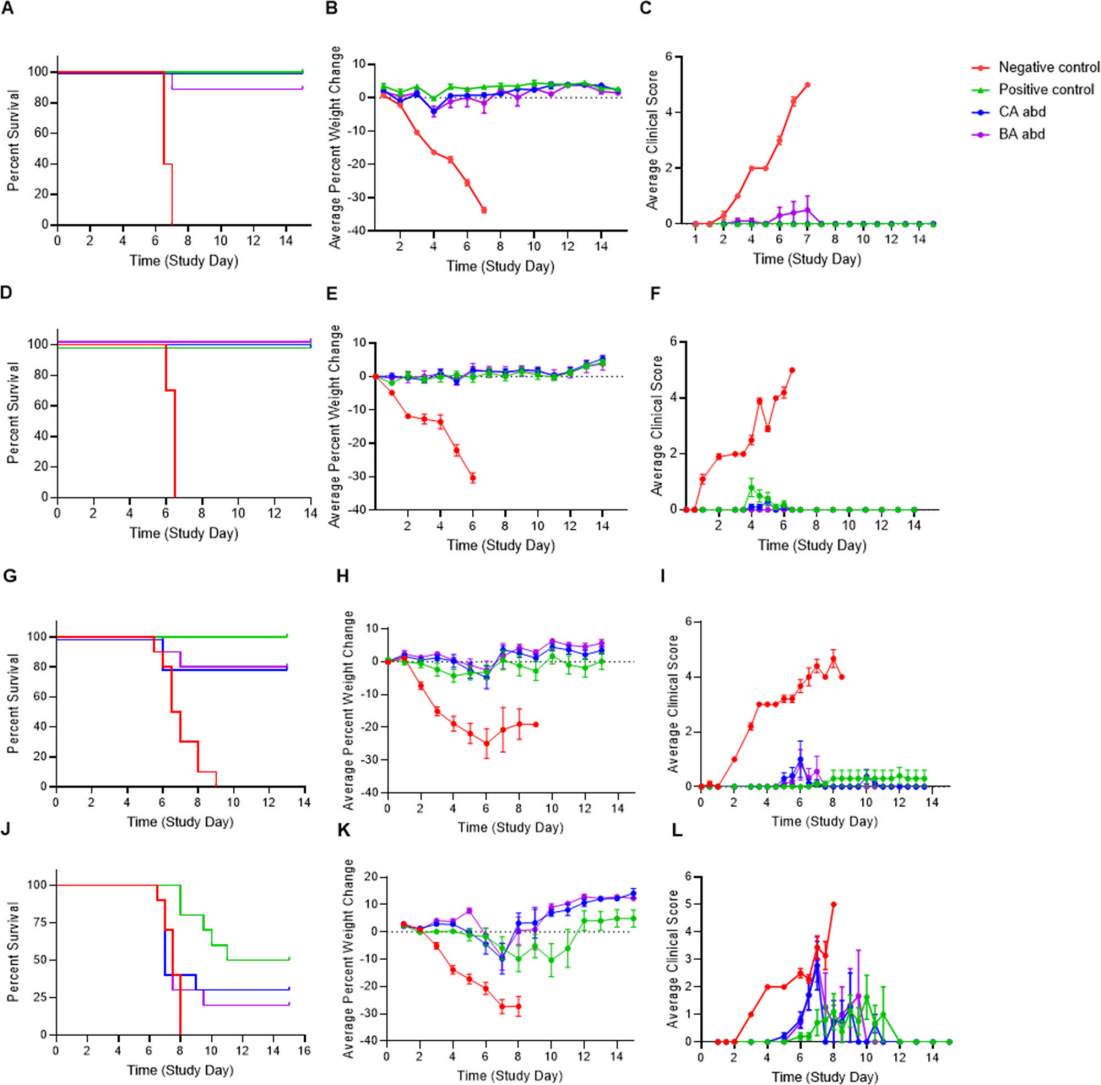
Efficacy of CA-abd and BA-abd against different subtypes of VEEV. Balb/c mice (*N* = 10/group) were challenged either subcutaneously (SC) in the rear footpad or via aerosol (AE) with a target dose of 1000 PFU with different subtypes of VEEV. (**A-C**) Mice were challenged SC with VEEV INH-9813 (epidemic IC subtype). (**D-F**), Mice were challenged SC with VEEV ZPC738 (enzootic ID subtype). (**G-I**) Mice were challenged AE with VEEV Trinidad donkey (epidemic IAB subtype). (**J-L**), Mice were challenged AE with VEEV INH-9813. One hour post-challenge, all mice received an administration of 200 µg of antibody. (**A, D, G, and J**) Survival curve; (**B, E, H, and K**) average percent weight change; (**C, F, I, and L**) average clinical score. Error bars represent standard error mean (SEM). Positive control for all experiments was anti-VEEV IgG 1A3B-7. Negative for the VEEV IC challenges was anti-EBOV IgG and for the VEEV ID and VEEV IAB challenge was E2C2-abd.

The first study evaluated the ability of the sdAbs to protect against a SC inoculation with the VEEV INH-9813 strain, a IC subtype isolated from a human infection in 1995 (24). Mice were inoculated with 1,000 PFU VEEV INH-9813 in the rear footpad, and therapeutic sdAb was administered intraperitoneally 1 h post-challenge. Control mice received either an irrelevant antibody (negative control) or the anti-VEEV IgG 1A3B-7 (positive control), which has previously shown efficacy in the mouse model against multiple strains of VEEV (25) and in the NHP model against VEEV TrD (18). Treatment with either CA-abd or BA-abd protected 90%–100% of the mice not only from lethality (*P* < 0.0001; Fig. 2A) but also from weight loss (*P* ≤ 0.002; Fig. 2B) and development of clinical signs of disease (*P* ≤ 0.005; Fig. 2C).

Since all subtypes of VEEV are capable of causing disease in humans (22), study 2 aimed to determine the breadth of CA- and BA-abd to protect against enzootic subtypes of VEEV. The VEEV ID subtype encompasses enzootic strains of VEEV that are mainly transmitted between the mosquito vector and the rodent reservoir host (26). Here, mice were SC inoculated with 1,000 PFU VEEV ZPC738 in the rear footpad, and again, therapeutic sdAb was administered intraperitoneally 1 h post-challenge. Similar to the IC subtype study, treatment with CA- or BA-abd was able to protect 100% of the mice not only from lethality (*P* < 0.0001; Fig. 2D) but also from weight loss (*P* ≤ 0.00008; Fig. 2E), development of clinical signs of disease (*P* ≤ 0.0001; Fig. 2F), and serum viremia (*P* ≤ 0.008; Fig. S2A). These data suggest that the two bivalent sdAbs have pan-anti-VEEV potential.

Previously, VEEV was developed as a threat agent due to the high rate of morbidity in humans with a subclinical to clinical symptom ratio of 1:1 (14, 27, 28). Past efforts to develop VEEV as an aerosol disseminated threat agent have resulted in the need to test any VEEV-specific medical countermeasures for the ability to protect against this challenge route. For this reason, studies 3 and 4 evaluated if CA- or BA-abd could protect mice against aerosol exposures to epizootic strains of VEEV (IAB and IC subtype, respectively). First, BALB/c mice were aerosol exposed to a target dose of 1,000 PFU VEEV TrD, an IAB strain initially isolated in 1943 (29) with a history of causing laboratory exposures (30, 31). Both CA- and BA-abd were able to protect 80% of mice from lethality (*P* ≤ 0.001; Fig. 2G), weight loss (*P* ≤ 0.01; Fig. 2H), development of clinical signs of disease (*P* ≤ 0.01; Fig. 2I), and serum viremia (*P* ≤ 0.02; Fig. S2B). Demonstrating protection against the IAB subtype, study 4 evaluated the sdAbs against the currently circulating epizootic subtype (IC) through an aerosol exposure with a target dose of 1,000 PFU VEEV INH-9813. Contrary to earlier experiments, the sdAbs were less effective at protecting mice against the VEEV IC aerosol exposure (Fig. 2J through L). Although there was no significant difference in the mean time-to-death between the negative control group (7.5 days) and the CA-abd-treated group (7.2 days), there was a significant reduction in the percent mortality (*P* = 0.03; Fig. 2J). Both the CA-abd- and BA-abd treated groups lost significantly less weight compared with the negative control group (*P* ≤ 0.004; Fig. 2K) and a significant delay in the development of clinical signs of disease compared with the negative control group (*P* ≤ 0.0006; Fig. 2L). Surprisingly, the 1A3B-7-positive control, which consistently provides 90-100% protection against aerosol exposure with VEEV TrD (Fig. 2G through I and (16, 25)) also failed to provide complete protection from the VEEV IC aerosol challenge (Fig. 2J). One explanation of the reduced protection of 1A3B-7-positive control against a VEEV IC aerosol is that 1A3B-7 has a 16-fold higher PRNT80 against VEEV IAB (0.049 µg/mL) than VEEV IC (0.781 µg/mL). Taken together, the data suggest that CA- and BA-abd bivalent sdAbs provide significant protection against multiple lineages and routes of exposures of VEEV.

### Cryo-EM structures and epitope identification

**Fig 3:**
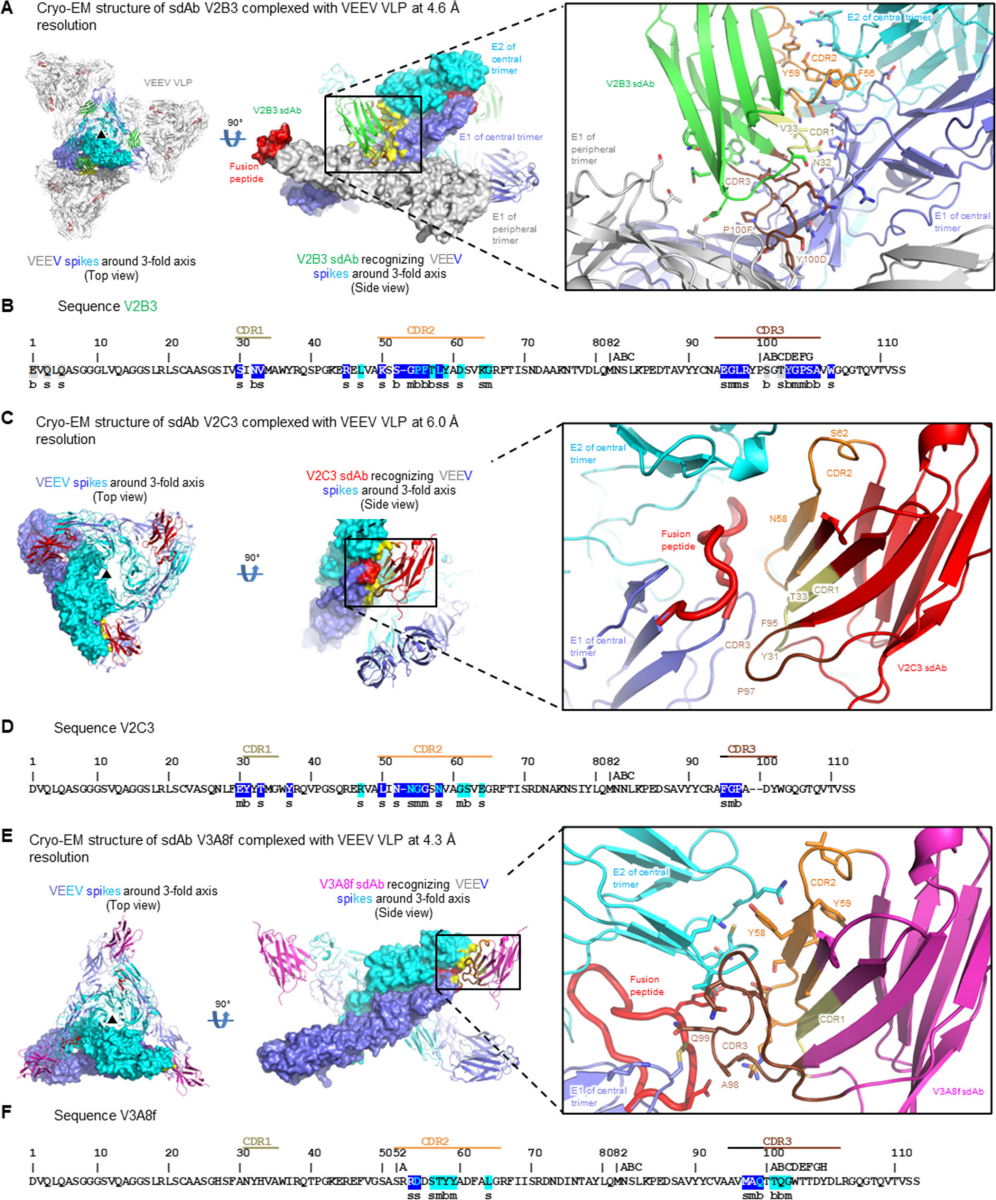
Cryo-EM structures of V2B3, V2C3, and V3A8f sdAbs in complex with VEEV VLP. (**A**) Cryo-EM structure of VEEV-V2B3 at 4.6 Å resolution. (left) Top view of four VEEV trimers shown arranged around an icosahedral threefold axis, with E1 subunit in slate blue and E2 subunit in cyan for the central spike, and gray for the other peripheral three spikes. The V2B3 sdAbs are shown in green ribbon, binding to E1E2 on the central spike and E1 on a peripheral spike (with location of fusion peptide highlighted in red). (middle) Enlarged side view, with same coloring as panel A, with CDR1 highlight in pale yellow, CDR2 in orange, and CDR3 in brown, and paratope on antibody surface highlighted in yellow. (**B**) Sequence of V2B3 (Kabat numbering – see Materials and Methods), with CDRs delineated and residues interacting the alphavirus trimer indexed by type of interaction (m, for mainchain; s, for sidechain; b, for both) and are highlighted by the color of the interacting subunit as defined in panel A. Those residues that interact with two subunits are dual colored. (**C**) Cryo-EM structure of VEEV-V2C3 at 6.0 Å resolution. (left) Top view colored as in panel **A**, for central trimer around icosahedral threefold with V2C3 in red. (middle and right) Side views colored as panel **A**, but with V2C3 in red. (**D**) Sequence of V2C3 displayed as in panel B. (**E**) Cryo-EM structure of VEEV-V3A8f at 4.3 Å resolution. (left) Top view colored as in panel A, for central trimer around icosahedral threefold with V3A8f in magenta. (middle and right) Side views colored as panel A, but with V3A8f in magenta. (**F**) Sequence of V3A8f displayed as in panel B.

Since both bivalent sdAbs provided protection from VEEV *in vivo*, structural insight of the binding epitopes of the three individual sdAb components of CA- and BA-abd against VEEV was sought to determine if each bound to unique or overlapping epitopes. Cryo-grids for the complex of sdAb V2B3, from the BA-abd, with VEEV virus-like particle (VLP) were prepared, and single particle cryogenic-electron microscopy (cryo-EM) data were collected on a Titan Krios. From a total of 41,401 particles, *ab initio* reconstruction for the VEEV VLP (strain TC83) utilizing icosahedral symmetry was performed. To visualize the sdAb V2B3, C1 symmetry expansion was performed with mask generation around four VLP spikes surrounding the icosahedral threefold axis, and signal subtraction and local refinement were performed within the mask to obtain a 4.6 Å reconstruction of the V2B3-VLP spike complex (Fig. 3A; Fig. S3; Table S2). V2B3 bound to the interface between three molecules: E1 and E2 of one trimeric spike [recognized by complementarity determining region 1 (CDR1), CDR2, and CDR3], as well as E1 from an adjacent trimeric spike (recognized by the sdAb N terminus as well as by CDR3) (Fig. 3B). This recognition appeared to lock trimers of the VLP together, suggesting a potential mechanism of blocking VLP disassembly as a means to inhibit virus entry.

For the structure of V2C3, from the CA-abd, with VEEV VLP, a similar approach was used, making cryo-grids for the complex of V2C3 sdAb and VEEV VLP and collecting single particle cryo-EM data. From a total of 31,101 particles, *ab initio* reconstruction for the VEEV VLP utilizing icosahedral symmetry was performed, and after masking, signal subtraction, and local refinement within the mask, a 6.0 Å reconstruction of the V2C3-VLP spike complex was determined (Fig. 3C; Fig. S4; Table S3). V2C3 bound to a site that overlapped the fusion loop, at the interface of E1 (recognized by CDR1-3) and E2 (recognized by CDR2) (Fig. 3D). This fusion peptide recognition likely disrupts the fusion of VLP and cellular membranes, thereby providing a potential mechanism for virus entry inhibition by this sdAb.

For the structure of V3A8f, present in both BA- and CA-abd, with VEEV VLP, cryo-grids for the complex of V3A8f and VEEV VLP were made, and single particle cryo-EM data were collected. From a total of 66,222 particles, *ab initio* reconstruction for the VEEV VLP utilizing icosahedral symmetry was performed, and after masking, signal subtraction and local refinement within the mask, a 4.3 Å reconstruction of the V3A8f-VLP spike complex was obtained (Fig. 3E; Fig. S24; Table S4). V3A8f also bound to a site that overlapped the fusion loop, at the interface of E1 (recognized by CDRs 2 and 3) and E2 (recognized by CDRs 2 and 3), likely inducing inhibition of the fusion mechanism, as a potential mechanism for inhibiting virus entry (Fig. 3F). Despite both V2C3 and V3A8f sdAbs recognizing sites that overlapped the fusion loop, the two nanobodies used different interactive surfaces and different recognition chemistries.

Having determined the structures of V2B3, V2C3, and V3A8f sdAbs in complex with VEEV VLP, we could now compare their epitopes to the location of the binding site for the LDLRAD3 receptor as well as to other VEEV-neutralizing antibodies whose structures have been determined in complex with VEEV VLP. V2C3 epitope partially overlapped the LDLRAD3 binding site, while both V2B3 and V3A8f epitopes were proximal but did not overlap (Fig. S3; Table S2). In addition, we compared the sdAb epitopes with those of SKV09, SKV16, SKT05, and SKT20, whose structures were previously defined (32); we observed overlap between the epitopes of SKT20 Ab and V2B3, V2C3, and V3A8f sdAbs (Fig. S4; Table S2). The epitope of SKT20 Ab also overlapped with the LDLRAD3-binding site (Table S2).

**Fig 4:**
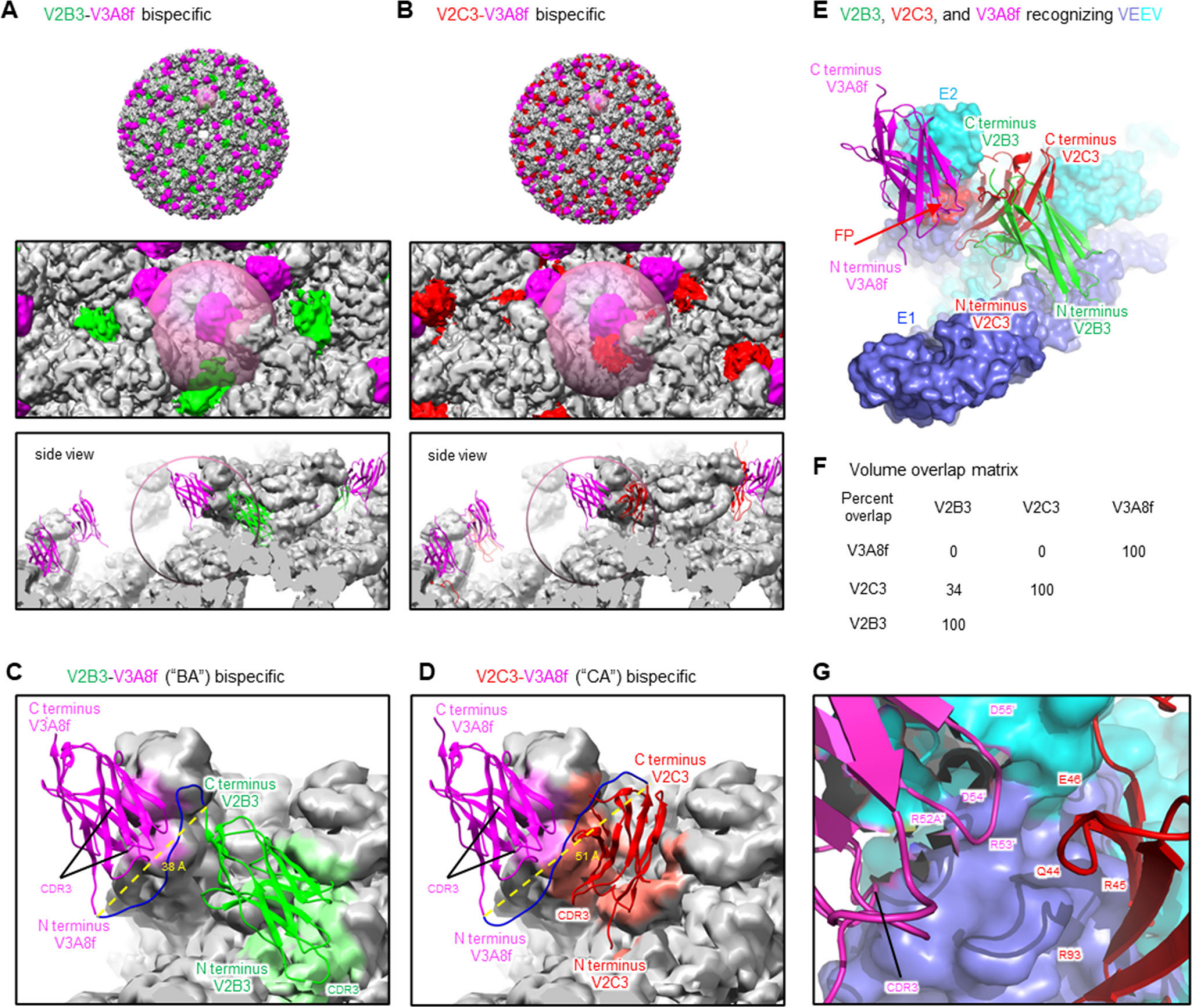
Bispecific sdAb linkage constraints. (**A**) V2B3 linked by AAA(GGGGS)_3_ to V3A8f. Top shows image of entire VLP positioning V2B3 (green) and V3A8f (magenta). Lower images show expanded views with 50 Å sphere around N-terminus of V3A8f. (**B**) V2C3 linked by AAA(GGGGS)_3_ to V3A8f. Top shows image of entire VLP positioning V2C3 (red) and V3A8f (magenta). Lower images show expanded views with 45 Å sphere around N-terminus of V3A8f. (**C**) Model of V2B3-V3A8f bispecific showing the single linkage that may enable bivalent recognition. (**D**) Model of V2C3-V3A8f bispecific showing the single linkage that may enable bivalent recognition. (**E**) Recognition of sdAbs on VEEV trimer. Top shows molecular models with trimer surface in slate blue and cyan for E1 and E2, respectively, and V2B3, V2C3, and V3A8f sdAbs in cartoon representation colored green, red, and magenta, respectively. (**F**) Volume overlap matrix, with percent overlap shown based on local superposition of VEEV E2. (**G**) Details of close contact between V2C3 and V3A8f.

Having determined the structures of V2B3, V2C3, and V3A8f sdAbs in complex with VEEV VLP, the structures were used to understand constraints to their linkage as bispecifics. Each of the separate structures of sdAbs was combined with the VEEV VLP to create a composite model (Fig. 4A), in which sdAbs V2B3 and V2C3 occupied overlapping positions. Calculation of overlap indicated 34% for V2B3 and V2C3, whereas V3A8f did not overlap either V2B3 or V2C3 (Fig. 4B). As there was no overlap with V3A8f, models of VEEV with this sdAb were examined, and either V2B3 (Fig. 4C) or V2C3 (Fig. 4D), specifically focusing on sdAbs that were within 60 Å of the N terminus of V3A8f, as these could then be covalently attached by a 20-residue linker. In both cases, only a single pair of sdAbs could be linked. For V2B3 linked to V3A8f, the calculated termini distance was 38 Å (Fig. 4E). For V2C3 linked to V3A8f, the calculated termini distance was 51 Å (Fig. 4F), although the physical distance was much closer, with side chains from framework regions 1 and 3 of V2C3 in close contact with side chains from the CDR2 region of V3A8f (Fig. 4G). Thus, structural modeling indicated two bispecific sdAbs were possible: V2B3-V3A8f, which comprised the BA-abd bivalent bispecific, and V2C3-V3A8f, which comprised the CA-abd bivalent.

### Breadth of binding and neutralization

**Fig 5:**
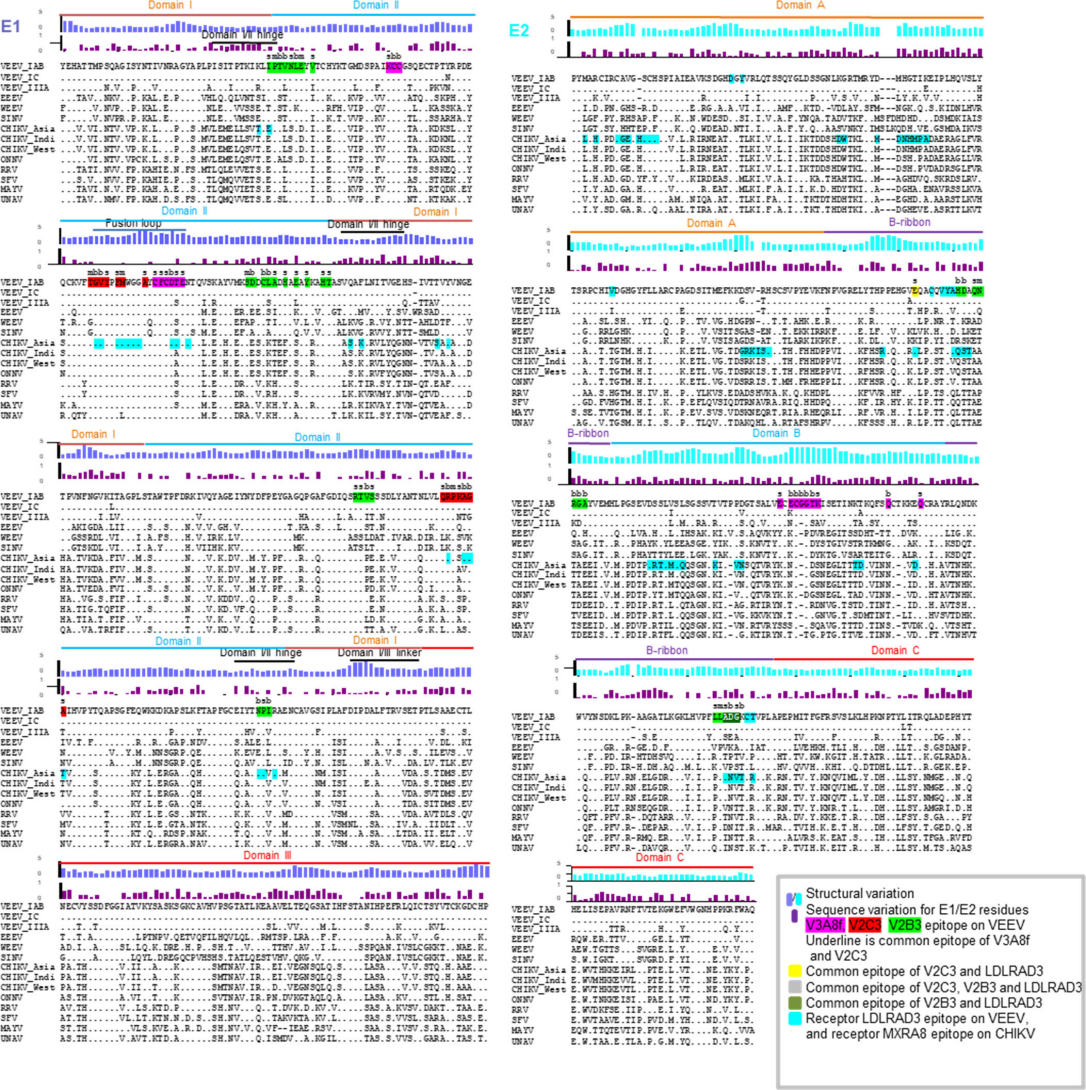
VEEV epitopes recognized by V3A8f, V2B3, and V2C3 sdAbs and their conservation within alphaviruses that infect humans. Alphavirus E1 (left) and E2 (right) sequences are shown, with structural and sequence variations plotted for each residue. V3A8f, V2C3, and V2B3 epitopes as well as virus receptors LDLRAD3 and MXRA8 epitopes are highlighted.

**Fig 6:**
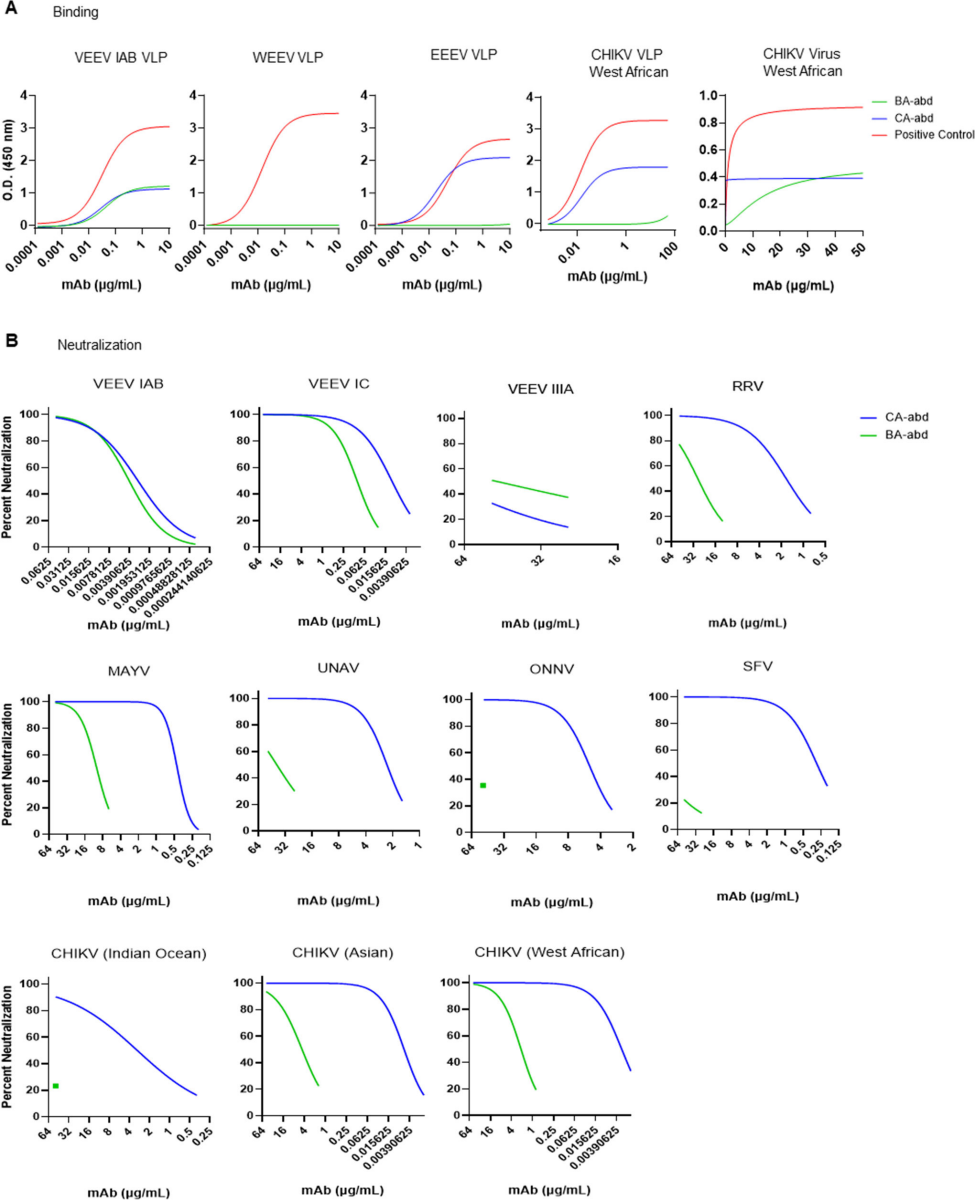
Broad alphavirus binding and neutralization of bivalent constructs linked with albumin binding domains. (**A**) ELISA binding curves for CA-abd and BA-abd against VEEV IAB VLP, VEEV IIIA E1-E2, WEEV VLP, EEEV VLP, and CHIKV VLP. Positive control for VLP was mAb IgG SKT05, and for CHIKV virus the positive control was sdAb CC3. (**B**) Neutralization curves of CA-abd and BA-abd.

With knowledge of the sdAbs VEEV VLP-binding epitopes, next the E1-E2 sequences of multiple alphaviruses were aligned to determine if the sdAbs bound conserved epitopes present in other alphaviruses (Fig. 5). For V3A8f, the epitope on E1 was highly conserved, but the region on E2 was not so conserved. Interactions with E2 involved both main chain and side chain interaction, suggesting the V3A8f would bind less well to divergent alphaviruses, with changes in E2. For V2B3, the recognized epitope was less conserved on E1 and quite variable on E2. For V2C3, the recognized portion of the epitope that overlaps with the fusion loop on E1 was conserved, while the recognized surface of domain II portion on E1 was less conserved. Based on the alignment, the CA-abd binds to more conserved epitopes than the BA-abd bivalent sdAb. Previously, we demonstrated that these sdAbs did not neutralize the other encephalitic alphaviruses western or eastern equine encephalitis virus (WEEV or EEEV, respectively) but did observe a reduction in plaque size against EEEV (6). Therefore, the ability of CA-abd and BA-abd to bind WEEV, EEEV, and the arthritogenic alphavirus chikungunya virus (CHIKV) VLP was evaluated since antibody binding with poor neutralization can still provide *in vivo* efficacy (18, 32–35). In line with previously published data (Table S1) (6), both antibodies were able to bind the VEEV IAB VLP (Fig. 6A). Not unexpectedly, neither sdAb bound to the WEEV VLP (Fig. 6A), which agrees with our previous observation when assessing neutralization against WEEV (6). Interestingly,the CA-abd had strong binding to EEEV VLP and CHIKV VLP (Fig. 6A), while BA-abd did not bind to EEEV VLP and bound weakly to both the CHIKV VLP and CHIKV virus compared with stronger binding by CA-abd (Fig. 6A).

**Fig 7:**
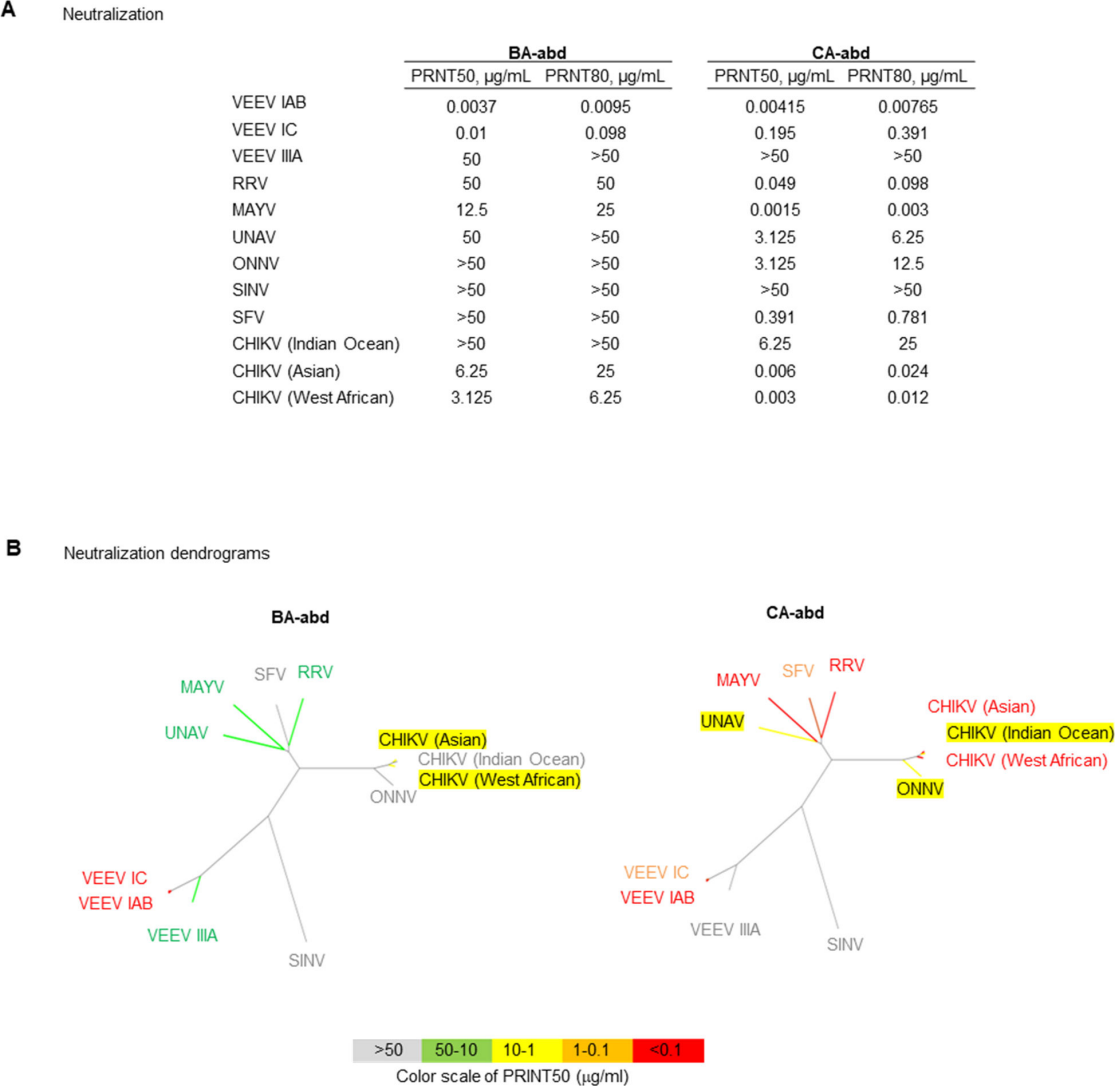
Comparing neutralization levels between the two bivalent sdAbs. (**A**) PRNT50 and PRNT80 neutralization levels of CA-abd and BA-abd. (**B**) Dendrogram colored by neutralization by BA-abd and CA-abd.

To determine the potential neutralizing breadth of the two bispecific sdAbs, the ability of the two sdAbs to neutralize multiple different alphaviruses, both encephalitic and arthritogenic, was evaluated. As expected, both sdAbs were potent neutralizers of VEEV IAB and VEEV IC strains (Fig. 6B and 7); however, the potency of neutralization was reduced for VEEV IC in comparison to VEEV IAB (≥10-fold difference) (Fig. 6B and 7). The reduced potency of the sdAbs against VEEV IC likely explains the reduced efficacy of these sdAbs against a VEEV IC aerosol challenge (Fig. 2J). While both bivalent sdAbs can neutralize multiple alphaviruses, CA-abd has the greatest breadth (10 of 12 viruses evaluated) and more potent neutralizing capabilities (Fig. 6 and 7) in comparison to BA-abd, which only neutralizes six of the viruses evaluated. Overall, these data suggest that the bispecific CA-abd antibody has the potential to be a pan-alphavirus therapeutic.

## DISCUSSION

SdAbs offer several advantages over conventional IgG including a reduced molecular mass allowing for better tissue penetration (36), increased thermostability (37), lower immunogenicity due to lack of Fc binding domain (2) and scalability for manufacturing due to flexibility in expression systems and purification methods (37). One disadvantage is a reduced half-life, a result of the smaller size, but this can be overcome with genetic fusions or PEGylation (3). By fusing the albumin binding domain (abd) to the bivalent sdAb constructs, we demonstrated we were able to maintain neutralization levels comparable to the bivalent constructs without the domain (Table S1) and have a tissue and serum half-life of ≥29 h (Fig. 1).

With the limited number of cases of VEEV per year, licensure of a therapeutic will likely occur under guidance of the FDA Animal Rule. Current guidance suggests challenge agents used in pivotal efficacy studies should replicate the etiological agent that causes disease in humans with no animal passaging and minimal cell culture passaging in the history of stock production (24). Frequently, vaccine and therapeutic efficacy studies utilize VEEV TC-83 or TrD strains as the challenge agent. While these strains are historically important, they are of the IAB subtype. There are two subtypes of VEEV that have been responsible for epidemic outbreaks: IAB and IC. Viral isolates of the IAB subtype have not caused outbreaks since the 1970s, instead strains from the IC subtype have been responsible for recent epidemic outbreaks (38). For that reason, we utilized VEEV INH-9813, a human isolate of the IC subtype with minimal cell culture passaging and no animal passaging (39), for our first efficacy study (Fig. 2A through C). A single dose of the bispecific sdAbs administered 1 h post-challenge was enough to protect the mice from a lethal SC VEEV IC challenge (Fig. 2A), providing protection from morbidity as well. Considering the significant sequence diversity across VEEV subtypes, it was important that the bispecific sdAbs were evaluated for efficacy against other lineages of VEEV, especially since all lineages are capable of causing disease in humans (22). Weaver et al. have hypothesized that mutations in the major antigenic sites of the E2 glycoprotein of enzootic VEEV strains result in emergence of epizootic strains responsible for some of the major human outbreaks (40, 41). Like the SC VEEV IC challenge, the two bispecific sdAbs were able to protect against both morbidity and mortality after a SC VEEV ID challenge (Fig. 2D through F). Since VEEV is on the CDC Select Agent list due to the potential of its use for nefarious purposes, we tested the ability of bispecific sdAbs to protect against an AE challenge against the two epizootic VEEV lineages as well. Both sdAbs protected 80% of the mice from a VEEV IAB AE challenge (Fig. 2G); however, less efficacy was observed against the VEEV IC AE challenge (Fig. 2J). Despite reduced ability to protect against lethality after a VEEV IC AE challenge, the sdAbs were able to significantly delay weight loss and onset of clinical signs of disease (Fig. 2K and L). *In vitro* neutralization assays found the CA-abd and BA-abd sdAbs neutralized VEEV IC less effectively than VEEV IAB (Fig. 6B). This result is somewhat expected as these sdAbs were originally identified based on binding and neutralization against VEEV TC-83, a VEEV IAB strain. Indeed, most VEEV-specific mAbs have been identified by panning against a VEEV IAB strain or were generated against a VEEV IAB antigen (32, 42–45); therefore, the gold standard for testing *in vivo* efficacy of VEEV-specific mAbs is to challenge with VEEV IAB (16, 25, 42, 46–48). For example, 1A3B-7, the mAb used as a positive control in these studies with demonstrated efficacy in NHP studies (18), provided complete protection against AE to VEEV IAB only provided partial protection against VEEV IC. Few mAbs have actually been evaluated against other lineages of VEEV by SC (25, 42) or AE challenge (17, 45). Nonetheless, CA- and BA-abd are potent neutralizers of VEEV IC. Future studies should test if efficacy could be increased with the addition of a second administration of sdAb ~3–4 days post-challenge. Our results highlight the need to expand testing and evaluation of new anti-VEEV Ab candidates against multiple VEEV subtypes by AE to identify the most efficacious protective anti-VEEV Abs.

The sdAbs have the potential for being a pan-alphavirus antibody. The V2C3 component of the CA antibody is in the same sequence family as the CHIKV CC3 sdAb that has been shown to neutralize CHIKV, RRV, and MAYV *in vitro* (5, 6). We expand on these results to determine how cross-protective the VEEV sdAbs are against not only other strains of VEEV but other alphaviruses such as CHIKV (Fig. 6). The CA-abd sdAb demonstrated the widest breadth and better neutralization against multiple alphaviruses compared with the BA-abd sdAb (Fig. 6). Based on sequence alignment, the difference in breadth of neutralization among VEEV strains by the sdAbs was due to recognizing epitopes that were conserved among all VEEV strains of interest, except for the more divergent VEEV IIIA strain, which had sequence differences in epitope residues for all three sdAbs (Fig. 5); with only the V3A8f epitope showing sequence variation with an E to K change in domain B of E2 for VEEV strain IC.

Additionally, although the different lineages of CHIKV are more closely related to each other than the other arthritogenic alphaviruses (Fig. 6C), there is sequence variation and structural variation across the CHIKV lineages at the binding sites for both sdAbs (Fig. 5), which could explain why the two sdAbs have different neutralization profiles against the CHIKV Indian Ocean lineage compared with the other two lineages (Fig. 6B). In follow-up studies, we would like to evaluate the CA-abd bispecific sdAbs ability to protect *in vivo* against other alphaviruses to demonstrate that these bispecifics could be utilized as a pan-alphavirus therapeutic. Although the ability to bind/neutralize multiple alphaviruses could decrease the likelihood of viral escape mutants, a cocktail of multiple pan-alphavirus binding/neutralizing Abs (32, 33, 49) may provide optimal coverage against alphaviruses while decreasing the prevalence of viral escape mutants, which could cause the therapeutic to no longer be efficacious.

## MATERIALS AND METHODS

### SdAb constructs and protein purification

The sdAb constructs utilized in this work are derivatives of constructs that have been previously published. The CA-abd and BA-abd are derivatives of the VEEV binding, bivalent sdAbs V2C3-V3A8f and V2B3-V3A8f, respectively appended with an albumin binding domain (abd) (6, 21). The protein sequence of the constructs is provided in Fig. S1.

Expression plasmids were transformed into ClearColi BL21(DE3), from Lucigen (Middleton, WI, USA), for protein production. Overnight cultures were started by inoculating either a freshly transformed colony, or 10 µL of a frozen cell stock, into 50 mL of LB Miller containing 100 µg/mL ampicillin and grown at 37°C overnight. The next day, the overnight culture was poured into 450 mL LB Miller (100 µg/mL ampicillin) in a 2-L baffled flask. Cultures were grown between 6 and 8 h at 30°C before the temperature was lowered to 25°C and the cultures induced by the addition of 0.5 mM IPTG, and grown overnight at 25°C.

After growth and induction, the solution was centrifuged to pellet the cells and the supernatant discarded. Pelleted cells from each 500 mL shake flask culture were resuspended in 14 mL of Tris-Sucrose buffer (100 mM Tris, 0.75M sucrose pH 7.5) by gently compressing them with a spatula. One milliliter of lysozyme (1 mg/mL) made up in Tris-Sucrose was added to the homogenized cells. Next, 28 mL of 1 mM EDTA was added drop-wise to the solution while the centrifuge tubes held in crushed ice were shaking on a rotating platform. After the addition of the EDTA, 0.25 mL of 5% deoxycholate was added, and the cells were gently swirled for another half hour. Finally, 1 mL of 0.5M MgCl_2_ was added to bind the free EDTA, and the mix was incubated for another 15 min prior to pelleting the spheroplasts. The supernatant was poured into a 50 mL conical tube containing 5 mL of 10× IMAC buffer (0.2 M Na_2_HPO_4_, 4 M NaCl, 0.2 M imidazole, pH 7.5) and 0.5 mL of Ni Sepharose (GE Healthcare) that had been washed in 1× IMAC. The mixture was tumbled at least 1 h at 4°C on a tube rotisserie. The resin was washed twice with 25 mL 1× IMAC buffer, and then the resin was incubated with 5 mL of PBS containing 0.2% Triton (TX)−114 for 1 h. Between 0.5 and 1 mL of the nickel resin was loaded onto a column and washed with PBS 0.2% TX-114 (10 mL) followed by two washes, 5 mL each, of 1× IMAC buffer to remove detergent. SdAb constructs were eluted from the nickel resin using 2 mL of elute buffer (1× IMAC plus 250 mM Imidazole) and collected into a 2 mL tube. The eluted material was applied straight onto a MEP HyperCel resin (Sartorius; 2 mL) column that had been washed with 1 mL of NaOH followed by endotoxin-free water and then endotoxin-free PBS. Once the sample was loaded, the MEP HyperCel column was washed with 30 mL of endotoxin-free PBS. The sdAb was eluted with 50 mM Na-Citrate pH 3.5, and sdAb containing fractions determined via monitoring OD280. The sample was neutralized using 10% vol of 1.0 M Tris-HCl pH 7.5. The neutralized elute from the MEP HyperCel was loaded onto a column containing 1 mL of fresh nickel resin that had been washed with endotoxin-free PBS. The sdAb was eluted slowly using elute buffer. For the final steps of purification, the FPLC system was equilibrated with endotoxin-free PBS using a SEC 650 column that has been kept endotoxin-free.

Purified sdAb was frozen for later use or filtered through a Mustang E filter (Pall), to remove residual lipids/endotoxin prior to freezing. The yield of the sdAb was determined by UV spectroscopy using a Nanodrop (Thermo). Extinction coefficients can be calculated by an on-line tool such as ExPASy (50) or approximated to be the same as a traditional IgG (1.4).

When purifying the CA-abd and BA-abd constructs, we utilized pyrogen-free pipette tips and pyrogen-free tubes in an effort to minimize contamination of the protein preparations with endotoxins. These constructs were purified on a SEC 650 10 × 300 column that was kept endotoxin-free. Before purification, the column and loop were cleaned by loading 1 mL of 1M NaOH into the loop and then manually injecting it onto the column and running 30 mL of endotoxin-free PBS through the column until the NaOH eluted. The loop was extensively rinsed with endotoxin-free water prior to loading the sample. Protein was collected into pyrogen-free microfuge tubes.

### Measuring endotoxin levels

The Charles River Endosafe-PTS instrument was used to measure endotoxin levels in sdAb preparations in accordance with manufacturer instructions.

### PK studies

Mice were administered 200 µg of either CA-abd or BA-abd antibody intraperitoneally. At 0.5, 2, 4, 8, and 24 h post-injection, sera and brains were collected (*n* = 4/time point) for a plaque reduction neutralization assay (PRNT). Sera and brains from two additional mice for each antibody evaluated were used as a negative control for the assays. Sera were diluted initially 1:10 in minimum essential medium (MEM) with 2% heat-inactivated (HI)-FBS, 1% HEPES, and 2% Pen/Strep, then serial 1:2 dilutions were made. VEEV-TrD stocks were diluted to a concentration of 2.0 × 1,000 PFU/mL and added 1:1 to the serially diluted samples (resulting in a 1:20 initial dilution) or control wells containing media alone for the virus only control. The entire mouse brain was homogenized and diluted 1:1 with virus. Samples were incubated overnight at 4°C. Six-well plates of VERO76 cells were infected with 0.1 mL of each serial dilution per well in duplicate, and plates were incubated at 37°C for 1 h. After 1-h incubation, cells were overlaid with 0.6% agarose in Basal Medium Eagle (BME) with 10% HI-FBS and 2% Pen/Strep, and incubated for ~24 h at 37°C, 5% CO_2_. A second overlay containing 0.6% agarose in BME with 10% HI-FBS, 2% Pen/Strep, and 4% of total volume neutral red vital stain was added to the wells and further incubated for 18–28 h to visualize plaques. Plaques were counted following incubation with stain overlay. The virus-only control was counted, and the endpoint titer was determined to be the highest dilution with ≥80% reduction (PRNT80) or ≥50% reduction (PRNT50) in the number of plaques observed relative to virus-only control wells. Neutralization titer was reported as the reciprocal of dilution for the serum. The limit of detection for serum was 1:20. VEEV ATCC hyperimmune mouse ascites fluid was utilized as a positive control. Normal human serum was used as a negative control.

### *In vivo* imaging system (IVIS)

Two milligrams of the CA-abd was labeled with VivoTag 680 XL (PerkinElmer) following the manufacturer’s protocol with a few adjustments. Briefly, we used a dye to protein ratio of ~3:1 for the labeling; 220 µg dye was brought up in DMSO and added to the CA-abd. After incubating for 30 min at room temperature, we added 20% vol of 0.1 M borate pH 9 and incubated for an additional 30 min. The reaction was stopped with 10 µl of 1 M ethanolamine. To separate the dye labeled CA-abd from free dye, the sample was added to a Zeba spin column (Pierce), which had been washed twice with endotoxin-free water by gravity, then three times with endotoxin-free PBS by centrifugation. The final dye to protein ratio was determined to be ~2 from the UV/visible spectra of the labeled material and calculated following the manufacturer’s protocol. Labeled CA-abd was quick frozen on dry ice and stored at −80°C until needed. PRNT values for the labeled CA-abd were confirmed to be similar to that of the unlabeled antibody before IVIS imaging on the IVIS Spectrum CT (PerkinElmer). Mice were administered 200 µg of the labeled CA-abd intraperitoneally (IP). At 0.5, 2, 4, 8, 24, and 29 h post-administration, the mice were anesthetized with isoflurane and imaged. Living Image Software (PerkinElmer) was used to analyze the images. Region of interest (ROI) of the same size was used to calculate the total radiant efficiency ([*P*/s]/[µW/cm²]) for both the whole body and head only.

### Efficacy studies

Mice were anesthetized with isoflurane and then subcutaneously (SC) infected with 1,000 PFU of either VEEV INH-9813 or VEEV ZPC738 in the rear footpad. For aerosol exposure to either VEEV TrD or VEEV INH-9813, mice were exposed to a target dose of 1,000 PFU using a whole-body exposure system. The aerosol challenge was generated using a Collison Nebulizer to produce a highly respirable aerosol (flow rate 7.5 ± 0.1 L/min). The system generates a target aerosol of 1 to 3 µm mass median aerodynamic diameter. The negative control (anti-EBOV IgG [VEEV IC challenges] or E2C2-abd [VEEV ID and VEEV IAB challenges]), the positive control (anti-VEEV IgG 1A3B-7), and sdAbs (CA-abd and BA-abd) mice received a single 200 µg administration IP 1 h post-infection. Mice were weighed daily and scored for clinical signs of disease at least once daily until reaching a clinical score of ≥3 and then were monitored twice daily. Clinical signs of disease included ruffled fur, hunched posture, lethargy, and neurological signs such as hindlimb paralysis. When animals reached a clinical score of 5, they were humanely euthanized. Survival curve statistics were determined by using the Log-rank (Mantel-Cox) test (GraphPad). A one-sided χ analysis was used to determine differences in overall percentage of survival in each group (GraphPad). Multiple Mann-Whitney tests using Holm-Šídák method and two-way ANOVA analysis were used to determine statistical differences in weight (GraphPad). Multiple Mann-Whitney tests using Holm-Šídák method were used to determine statistical differences in clinical score (GraphPad).

### Viruses

VEEV INH9813 (IC strain) stock was passaged three times on VERO cells. VEEV Trinidad donkey (IAB strain) stock was received from DynPort Vaccine Company (DVC) and prepared by Commonwealth Biotechnologies Inc. VEEV ZPC738 (ID strain) stock was passaged once on BHK cells. VEEV TC83 (vaccine strain) was obtained from BEI (NR-63) and passaged one additional time. The following reagent was obtained through BEI Resources, NIAID, NIH and passaged 1×: VEEV TC83 (IAB vaccine strain; NR-63), UNAV Mac150 (NR-49912), MAYV Guyane (NR-49911), RRV Raratonga (NR-51647), CHIKV LR 2006-OPY1 (Indian Ocean lineage; NR-49741), CHKV PM 2951 (West African lineage; NR-49905), and ONNV UgmP30 (NR-51661). These viruses were obtained from WRCEVA NIAID and passaged 1×: SINV Eg339 (TVP21615) and SFV (TVP20172). VEEV Mucambo (IIIA) was passaged 4× on BHK cells. CHIKV 15561 (Asian lineage) has an unknown passage history.

### Serum viremia plaque assay

Sera were diluted in MEM with 2% HI-FBS, 1% HEPES, and 2% Pen/Strep, and serial (1:10) dilutions were made. Six-well plates of VERO76 cells were infected with 0.2 mL of each serial dilution per well in duplicate wells, and plates were incubated at 37°C for 1 h. After 1-h incubation, cells were overlaid with 0.6% agarose in BME with 10% HI-FBS, and 2% Pen/Strep, and incubated for ~ 24 h at 37°C, 5% CO_2_. A second overlay containing 0.6% agarose in BME with 10% HI-FBS, 2% Pen/Strep, and 4% of total volume neutral red vital stain was added to the wells, and plates were further incubated for 18–28 h for visualization of plaques. Plaques were counted following incubation with stain overlay. Virus stock was utilized as a positive control, and media were used only as a negative control. The limit of detection of the assay is 2.5E + 01 PFU/mL.

### Cryo-EM sample preparation, data collection, and processing

VEEV-V2B3, VEEV-V2C3, and VEEV-V3A8f complexes were prepared by mixing VEEV VLP (TC83 strain) with the sdAb at a 1:2 molar ratio to a final total protein concentration of 0.4 mg/mL. Quantifoil R2/2 mesh 200, 2 nm carbon gold grids were glow-discharged in PELCO easiGlow glow discharge unit (Ted Pella, Inc.) set to air pressure of 0.40 bar, current of 10 mA, and duration of 10 s.

Due to a low concentration of the VLP complex stock solution, before freezing, the grid was saturated by three consecutive applications of 2.7 µL of the sample, followed by edge blotting. The sample was then vitrified in liquid ethane using a FEI Vitrobot Mark IV set to 4°C chamber temperature, 95% chamber humidity, blot time of 1.5 or 2 s, and a blot force of −5. Cryo-EM data were collected on a Titan Krios G1 transmission electron microscope (FEI Company, Inc.) operating at 300 keV, equipped with an Apollo direct electron detection device (Direct Electron, Inc.). Exposures were taken in a movie mode, at a pixel size of 1.11 Å/pix, with a total dose of 40 e^–^/Å^2^ fractionated over 40 raw frames, defocus values set to cycle between −0.75 and −2 mm with SerialEM (51).

All data sets were processed with the cryoSPARC 4.4 software package (52). Movies were aligned and dose-weighted with patch motion correction, and the micrograph contrast transfer function parameters were gaged with patch CTF estimation. Curate exposures were used to filter out bad micrographs. Particles were picked with the blob picker, cleaned with inspect particle picks, extracted from micrographs, subjected to 2D classification, and the best classes were selected. *Ab initio* reconstruction, heterogeneous, homogeneous, and non-uniform 3D refinements were run with the "I" symmetry imposed. To improve the resolution of the final map, particles were symmetry expanded, and local refinement was performed using a mask covering the region of four spikes clustered around the icosahedral threefold axis and electron density corresponding to the nanobodies. DeepEMhancer (53) was used for map post-processing.

### Model building, refinement, and structural analysis

Homology models of the V2B3, V2C3, and V3A8f sdAbs were generated with the Alphafold2 algorithm (54) incorporated into ColabFold (55) server (https://colab.research.google.com/github/sokrypton/ColabFold/blob/main/beta/AlphaFold2_advanced.ipynb). To obtain initial atomic models of the complexes, previously deposited VEEV structure (PDB: 7FFE) (56) and *in silico* generated models of the sdAbs were docked into corresponding cryo-EM maps with UCSF Chimera (57). The structures were refined by rounds of real-space refinement in Phenix (58) alternating with model building in Coot (59). Structure validation was performed with Molprobity (60, 61) built-in into the Phenix suite and the wwPDB validation service (https://validate-rcsb-2.wwpdb.org/). The analysis of VLP-sdAbs interfaces was done with PISA (62) incorporated into the PDBePISA service (https://www.ebi.ac.uk/pdbe/pisa/pistart.html). The assessment of cryo-EM reconstruction quality and model refinement statistics is summarized in Fig. S3 to S5, and Tables S2 to S4.

Local superpositions of the structures were done in Coot. Complementarity-determining regions of the sdAbs were mapped with the abYsis key annotation service (http://www.abysis.org/abysis/index.html). Figures were generated with PyMOL (Schrodinger; http://www.pymol.org) and UCSF Chimera. Structural variation was calculated in Pymol after aligning the structures, sequence variation was calculated as normalized Shannon’s entropy among the sequences and done with in-house script.

### Binding

#### VLP

96-well ELISA plates (Nunc) were coated with 2 µg/mL of virus-like particles (VLPs; WEEV CBA87 strain, EEEV VLP PE6 strain, VEEV IAB TC83 strain, CHIKV VLP 37997 strain) in PBS, pH 7.4, incubated at 4°C overnight, and blocked with PBS containing 5% skim milk (Difco) and 2% BSA (Fisher Scientific) (blocking buffer) at room temperature for 1 h. Each mAb was serially diluted with 5% skim milk and 2% BSA in PBS with 0.05% Tween-20 (PBST) dilution buffer and was added to the plate and incubated at room temperature for 1 h. After washing, horseradish peroxidase (HRP)-conjugated VHH domain-specific goat anti-alpaca IgG (Jackson ImmunoResearch Laboratories; cat#128-035-232) was added and incubated at room temperature for 1 h. Tetramethylbenzidine (TMB, SeraCare) HRP substrate was added to each well, and the reaction was stopped after 10 min by adding 1 M H_2_SO_4_. The absorbance was measured at 450 nm.

#### Virus

96-well ELISA plates (Thermo; Immulon 2HB) were coated with 3 µg/mL of sucrose purified CHIKV strain PM2951 in PBS, incubated at 4°C overnight. Next, coated plates were fixed with 10% formalin for 1 h. After the fixative was removed, plates were washed 3× with PBS + 0.02% Tween-20 (PBST), and blocked with Neptune buffer + 3% normal goat serum at ambient temperature for 5 h. After blocking, plates were washed 3× with PBST. Samples were diluted twofold in blocking buffer starting at 50 µg/mL. Plates were incubated overnight at 4°C. Following incubation, plates were washed 3× with PBST, a secondary anti-alpaca horseradish peroxidase-conjugated antibody (Jackson ImmunoResearch Laboratories; cat#128-035-232) diluted in blocking buffer was added, and plates were incubated for 1 h at ambient temperature. Next, plates were washed 3× with PBST, TMB substrate was added, and plates were incubated for ~5 min at ambient temperature. Finally, the reaction was stopped with Stop Solution, and absorbance was read using a Spectramax M5 instrument set at 450 nm.

### Plaque reduction neutralization assay (PRNT)

#### All PRNTs except ONNV

CA-abd and BA-abd were diluted at an initial concentration of 100 µg/mL in MEM with 2% HI-FBS, 1% HEPES, and 2% Pen/Strep, and serial 1:2 dilutions were made. Virus stocks were diluted to a concentration of 2,000 PFU/mL and added 1:1 to the serially diluted samples (resulting in a 50 µg/mL initial dilution) or control wells containing media alone for the virus only control. Samples were incubated overnight at 4°C. Six-well plates of Vero 76 cells were infected with 0.1 mL of each serial dilution per well in duplicate, and plates were incubated at 37°C for 1 h. After 1 h incubation, cells were overlaid with 0.6% agarose in BME with 10% HI-FBS and 2% Pen/Strep and incubated for ~24 h at 37°C, 5% CO_2_. A second overlay containing 0.6% agarose in BME with 10% HI-FBS, 2% Pen/Strep, and 4% of total volume neutral red vital stain was added to the wells and further incubated for 18–28 h to visualize plaques. Plaques were counted following incubation with stain overlay. The virus-only control was counted, and the endpoint titer was determined to be the highest dilution with ≥80% reduction (PRNT80) or ≥50% reduction (PRNT50) in the number of plaques observed relative to virus-only control wells. Neutralization titer was reported as the reciprocal of dilution for the serum. The limit of detection for serum was 50 µg/mL. Virus-specific ATCC hyperimmune mouse ascites fluid was utilized as a positive control, except for UNAV, as an UNAV-specific hyperimmune mouse ascites fluid was not commercially available. For UNAV, a CHIKV-specific ATCC hyperimmune mouse ascites fluid was utilized. ACVE sdAb was used as a negative control.

#### ONNV PRNT

CA-abd and BA-abd were diluted at an initial concentration of 100 µg/mL in MEM with 2% HI-FBS, 2% HEPES, 2% L-glutamine/GlutaMAX, and 2% Pen/Strep and then serially diluted (1:2). Virus stocks were diluted to a concentration of 2.0 × 10^3^ PFU/mL and added 1:1 to the serially diluted samples (resulting in a 50 µg/mL initial dilution) or control wells containing media alone for the virus only control. Samples were incubated overnight at 4°C. Six-well plates of Vero CCL-81 cells were infected with 0.1 mL of each serial dilution per well in duplicate, and plates were incubated at 37°C for 1 h. After 1 h incubation, cells were overlaid with 0.6% agarose in BME with 20% HI-FBS, 2% non-essential amino acids, 2% L-glutamine/GlutaMAX, and 2% Pen/Strep, and incubated for ~48 h at 37°C, 5% CO_2_. A second overlay containing 0.6% agarose in BME with 10% HI-FBS, 2% non-essential amino acids, 2% L-glutamine/GlutaMAX, 2% Pen/Strep, and 4% of total volume neutral red vital stain was added to wells, and the plates were further incubated for 18–28 h for visualization of plaques. Plaques were counted following incubation with stain overlay. The virus-only control was counted, and the endpoint titer of test sera was determined to be the highest dilution with ≥80% reduction (PRNT80) or ≥50% reduction (PRNT50) in the number of plaques observed relative to virus-only control wells. Neutralization titer was reported as the reciprocal of dilution for the serum. The limit of detection for serum was 50 µg/mL. A CHIKV-specific ATCC hyperimmune mouse ascites fluid was utilized for a positive control as an ONNV-specific hyperimmune mouse ascites fluid was not commercially available. ACVE sdAb was used as a negative control.

## Data Availability

The data presented in this study are provided within this article and available upon request from the corresponding authors. The cryo-EM maps and structure coordinates for the nanobody-VEEV VLP complexes were deposited to the EMDB and PDB databases under accession codes EMD-49740 and 9NRX (V2B3-VEEV), EMD-49741 and 9NRY (V2C3-VEEV) and EMD-49742 and 9NRZ (V3A8f-VEEV).

## SUPPLEMENTAL MATERIAL

Supplemental material jvi.01875-25-s0001.pdf Figures S1 to S7; Tables S1 to S6.

ASM does not own the copyrights to Supplemental Material that may be linked to, or accessed through, an article. The authors have granted ASM a non-exclusive, world-wide license to publish the Supplemental Material files. Please contact the corresponding author directly for reuse.
