# Supplementary material for: Demonstration of in vivo efficacy, cryo-EM-epitope identification, and breadth of two anti-alphavirus bispecific single domain antibodies
Source: J Virol. 2025 Dec 16;100(1):e01875-25. doi: 10.1128/jvi.01875-25 (PMC12817928; doi:10.1128/jvi.01875-25)
Supplement: Supplemental material — Figures S1 to S7; Tables S1 to S6. [file jvi.01875-25-s0001.pdf]

Table S1 Neutralization of VEEV TC-83 by plaque reduction neutralization assay for bivalent constructs vs bivalent constructs linked with albumin binding domains

SdAb construct	PRNT50, µg/mL	PRNT80, µg/mL
CA (V2C3-V3A8f)	0.002±0.0011	0.00675±0.0004
CA-abd	0.00415±0.0022	0.00765±0.0006
CA-alb1	0.005±0.0003	0.0139±0.0001
BA (V2B3-V3A8f)	0.0034±0.0001	0.0098±0.0006
BA-abd	0.0037±0.0013	0.0095+0.0001
BA-alb1	0.0069±0.0008	0.0175±0.0040

>BAabd

CDR1 (30–35), CDR2 (50–65), CDR3 (95–102); Kabat numbering 1 … 82 ABC … 100 ABCDEFG … 110

EVQLQASGGGLVQAGGSLRLSCAASGSIVSINVMAWYRQSPGKERELVAKSS-GPFTLYADSVKGRFTISNDAAKNTVDLQMNSLKPEDTAVYYCNAEGLRYPSGTYGPSAVWGQGTQVTVSS

1 … 20

AAAGGGGSGGGGSGGGGSGS

1′ … 52′A … 82′ABC … 100′ABCDEFGH … 110′

DVQLQASGGGSVQAGGSLRLSCAASGHSFANYHVAWIRQTPGKEREFVGSASRRDDSTYYADFALGRFIISRDNDINTAYLQMNSLKPEDSAVYYCVAAVMAQTTQGWTTDYDLRGQGTQVTVSS

1 … 20

AAAGGGGSGGGGSGGGGSGS

1″ … 59″

QHDEAVDANSLAEAKVLANRELDKYGVSDYYKNLINNAKTVEGVKALIDEILAALPGGA

>CAabd

CDR1, CDR2, CDR3; Kabat numbering 1 … 82 ABC … 100 … 110

DVQLQASGGGSVQAGGSLRLSCVASQNLFEYYTMGWYRQVPGSQRERVALIN-NGGSNVAGSVEGRFTISKDNAKNSIYLQMNNLKPEDSAVYYCRAFGPA--DYWGQGTQVTVSS

1 … 20

AAAGGGGSGGGGSGGGGSGS

1′ … 52′A … 82′ABC … 100′ABCDEFGH … 110′

DVQLQASGGGSVQAGGSLRLSCAASGHSFANYHVAWIRQTPGKEREFVGSASRRDDSTYYADFALGRFIISRDNDINTAYLQMNSLKPEDSAVYYCVAAVMAQTTQGWTTDYDLRGQGTQVTVSS

1 … 20

AAAGGGGSGGGGSGGGGSGS

1″ … 59″

QHDEAVDANSLAEAKVLANRELDKYGVSDYYKNLINNAKTVEGVKALIDEILAALPGGA

Fig S1 Amino acid sequences of CA-abd and BA-abd bispecific sdAbs. Within the constructs single sdAbs V2B3, V2C3 and V3A8f are colored green, red, and magenta, respectively. The linkers are black and the albumin binding domains are yellow. Bispecific constructs have Kabat numbering as in figure 3 with the first sdAb and the linkers numbered by regular numbers, the second sdAb numbered by primed numbers, and albumin binding domains numbered by double-primed numbers.

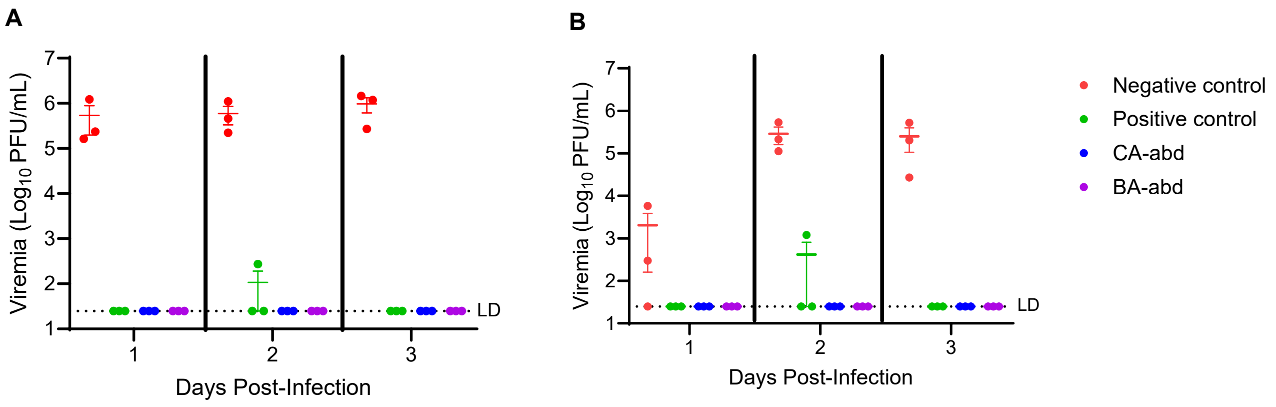

Fig S2 Bivalent sdAbs are able to dramatically reduce viremia after VEEV ID SC and VEEV IAB AE challenge. Balb/c mice (n=10/group) were challenged either subcutaneiously (SC) in the rear footpad or via aerosol (AE) with a target dose of 1000 PFU with different subtypes of VEEV. (**A**) Mice were challenged SC with VEEV ZPC738. (**B**) Mice were challenged AE with VEEV Trinidad donkey (epidemic IAB subtype). One hour post-challenge all mice received an administration of 200 µg of antibody. At days 1-3 post-challenge, three mice were bleed and serum was collected for a standard plaque assay. Error bars represent standard error mean (SEM). Positive control was anti-VEEV IgG 1A3B-7 and negative was E2C2-abd.

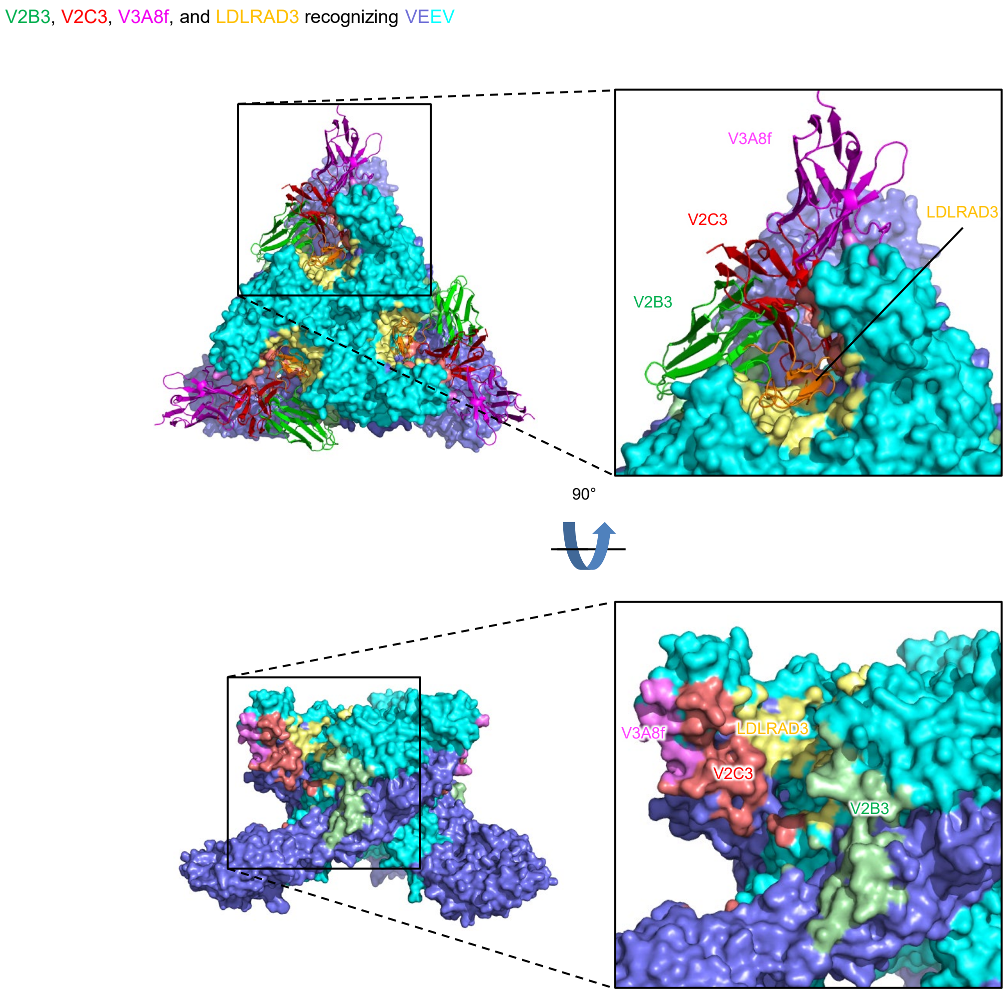

Fig S3 Footprints of sdAbs and Ldlrad3 receptor on VEEV VLP. sdAbs V2B3, V2C3 and V3A8f are colored green, red, and magenta, respectively. Ldlrad3 receptor (PDB: 7N1H) is colored yellow. The epitope of V2C3 partially overlaps with the Ldlrad3 receptor binding site.

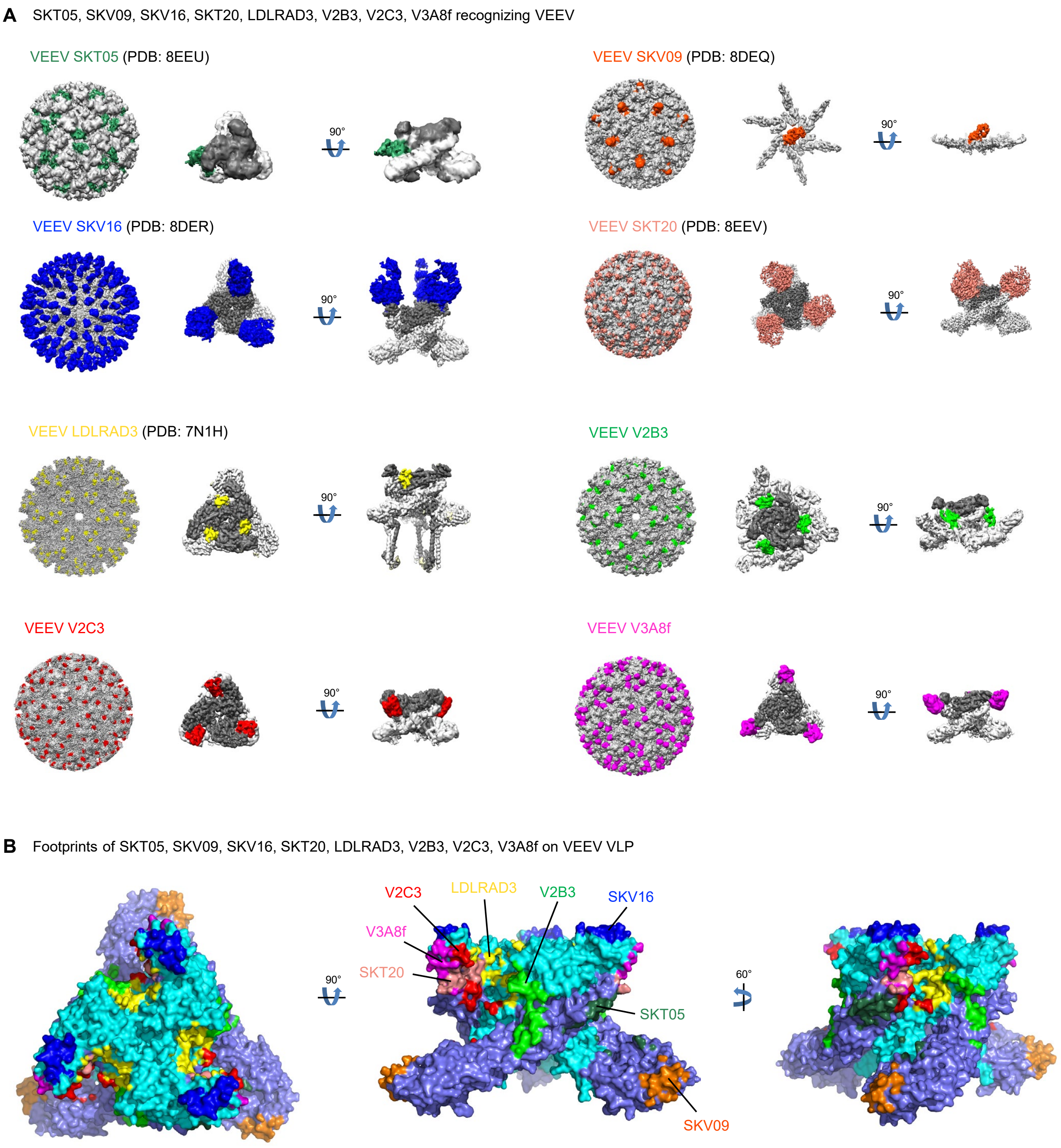

Fig S4 Footprints of various VEEV VLP-Ab and VEEV VLP-sdAb complexes. (**A**) The structures of VEEV VLP-LDLRAD3, VEEV VLP-Ab and VEEV VLP-sdAb complexes. VEEV VLP is shown in gray, and LDLRAD3, Abs, sdAbs are shown in various colors. (**B**) Enlarged view of VEEV spike showing the footprints of SKT05, SKV09, SKV16, SKT20 Abs; V2B3, V2C3, V3A8f sdAbs; and LDLRAD3 receptor. Epitope of SKT20 overlaps with the epitopes of V2B3, V2C3 and V3A8f. The binding site of LDLRAD3 receptor overlaps with the epitopes of SKT20 Ab and V2C3 sdAb.

Table S2 Contact residues in various VEEV VLP-Ab and VEEV VLP-sdAb complexes. Overlapping epitopes are highlighted: SKT20-V2B3 (green), SKT20-V2C3 (red), SKT20, and V3A8f (magenta). The footprint of LDLRAD3 receptor overlaps with the epitopes of SKT20, and V2C3 (yellow).

VEEV-Ab/sdAb	Epitope (10 A^2 cutoff)	Paratope (10 A^2 cutoff)	Reference
VEEV-V2B3	**$E1_1$:** I39, T41, V42, N43, L44, D112, L115, H118, E120, Y122, K123, H125, R206, T207, V208, S209, N264, P265, I266	S30, N32, V33, R45, K50, S52, G54, P55, F56, L58, E95, G96, L97, R98, Y100D, G100E, P100F, S100G, A101, W103	This work
	**$E2_1$:** D157, Q159, R161, G162, A163, L260, L261, A262, D263, K265	L47, P55, F56, T57, L58, Y59, D61, K64, G65	
	**$E1_2$:** I39, T41, V42, L44, T126, T207, S209	E1, Q3, Q5, S100A, T100C, Y100D	
VEEV-V2C3	**E1:** G83, Y85, F87, D97, T98, Q222, R223, K225, A226, G227	E30, Y31, T33, Y37, L50, N52, N54, G55, G56, N58, F95, G96, P97	This work
	**E2:** R18, H28, K222, K223, E224, R227	R47, N54, G55, N21, G61, S62, E64	
VEEV-V3A8f	**E1:** C63, F95, C96 E99	R53, D54, M97, A98, Q99	This work
	**E2:** E201, C202, G203, G204, T205, K206, Q219, Q225	D54, S56, T57, Y58, Y59, L64, Q99, T100A, Q100B	
VEEV-SKT05 (PDB: 8EEU)	**$E1_1$:** S65, E67, C68, P70, Y72, D75, E76, Q77, C78, K79, V80, A215, N216	**HC:** D31, W33, D53, R94, D96, R97, T98, S99, C100, R100A, R100B, G100C, C100E, H101	Sutton et.al.
	**$E1_1$:** K79, V80, T82, T98, E99, R223	**LC:** F31, Y49, D50, E53, L54, G56, G57, S60	
	**$E1_2$:** K340, T389, H390	**LC:** T5, L11, R24, T69, D70	
VEEV-SKT20 (PDB: 8EEV)	**E1:** F87, M88, W89, Y93, F95, D97, G227	**HC:** F50, H52, T54, K56, F58, C98, S99, D100, T100A, Y100D	Sutton et.al.
	**E2:** D94, H156, D157, A158, Q159	**HC:** S26, G27, S28, D30, T73, S74, R75, D76	
	**E1:** C63, M88, F95, E99	**LC:** H27D, S27E, N28, Y32, A91, I92, F94, W96	
VEEV-SKV09 (PDB: 8DEQ)	**E1:** A298, E300, K319, S321, Q349, G350, S351	**HC:** Y33, Y52, T54, D56, N58, S96, G97, D98, W99	Sutton et.al.
		**LC:** S30, W32, Y91, I92, S93, R94	
	**E1:** S323, K324, S325, E345, T347, E348, S351		
VEEV-SKV16 (PDB: 8DER)	**E2:** S180, T189, P190, D192, G193, I211, N212, K213, T214, K215, Q216, S218	**HC:** R28, D31, F32, H33, H52A, V55, D95, P97, Y98, W99, G100, Y100B	Sutton et.al.
**VEEV-receptor**	**VEEV interface (10 A^2 cutoff)**	**Receptor interface (10 A^2 cutoff)**	
LDLRAD3 (PDB: 7N1H)	**$E1_1$:** F87, W89, G91, A92	M36, S38, N39, G40	Basore et.al.
	**$E2_1$:** V24, S26, C27, H28, H71, S176, S177, K223	S38, N39, R41, C42, P44, W47, F56, D57, K62	
	**$E2_2$:** L5, Y44, G63, R64, V93, D94, E148, V153, A155, H156, D157, A158, A262, D263, G264, K265	E28, N30, I31, P32, G33, N34, P44, G45, A46, W47, G51, L52	

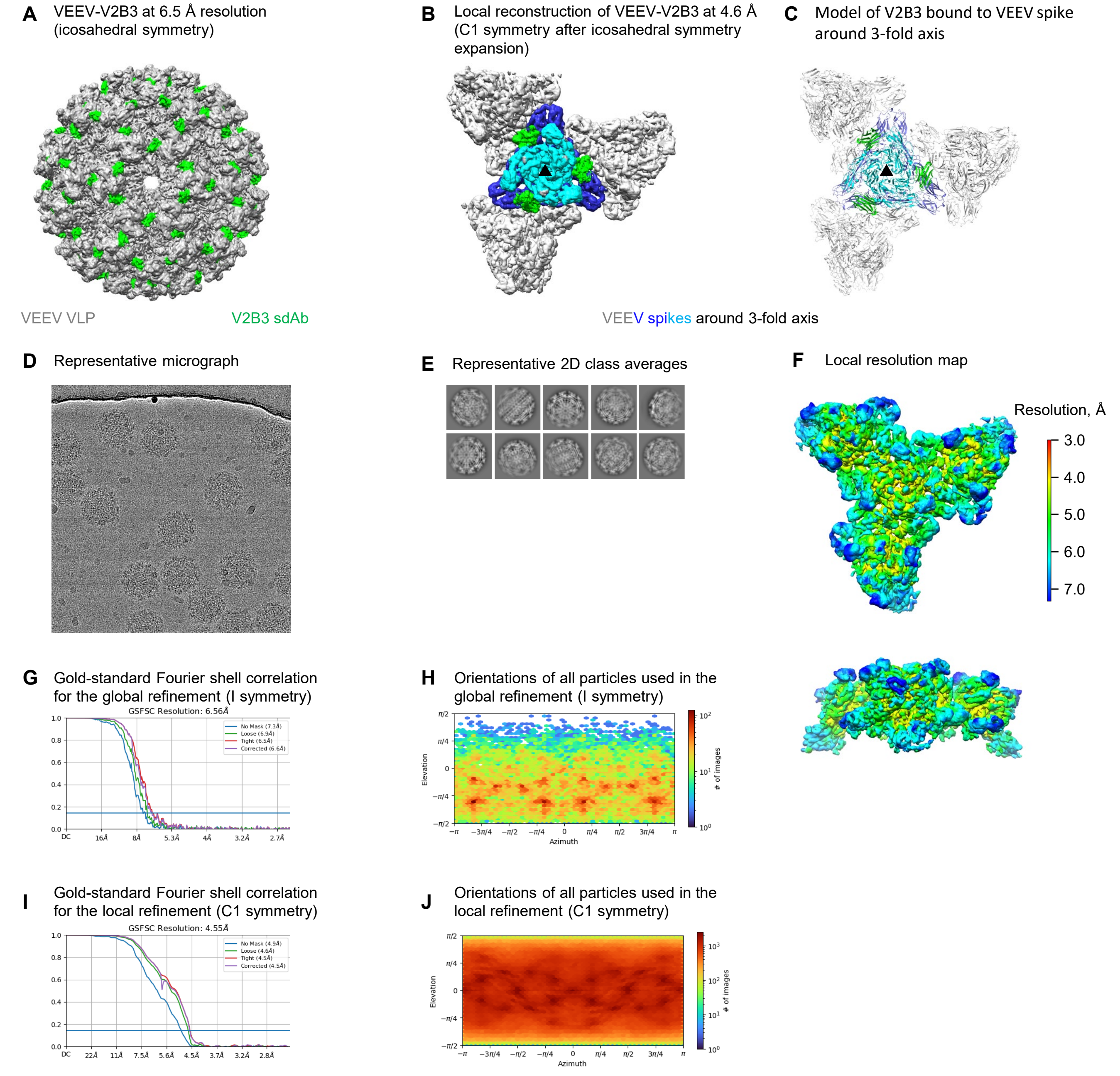

Fig S5 Cryo-EM structure of V2B3 sdAb in complex with VEEV VLP. (**A**) Cryo-EM reconstruction of VEEV-V2B3 complex at 6.5 Å resolution. V2B3 is shown in green. (**B**) Local reconstruction of four VEEV trimers after symmetry expansion arranged around an icosahedral 3-fold axis, with E1 subunit in slate blue and E2 subunit in cyan for the central spike, and grey for the other peripheral three spikes and V2B3 in green. (**C**) Model of VEEV-V2B3 complex colored as panel **B.** (**D**) representative micrograph. (**E**) Representative 2D class averages. (**F**) Local resolution map. (**G**) Gold-standard Fourier shell correlation for the entire complex after global refinement. (**H**) Heatmap showing the orientations of all particles used in the global refinement. (**I**) Gold-standard Fourier shell correlation for the four spikes arranged around icosahedral 3-fold axis after symmetry expansion and local refinement. (**J**) Heatmap showing the orientations of all particles used in local refinement.

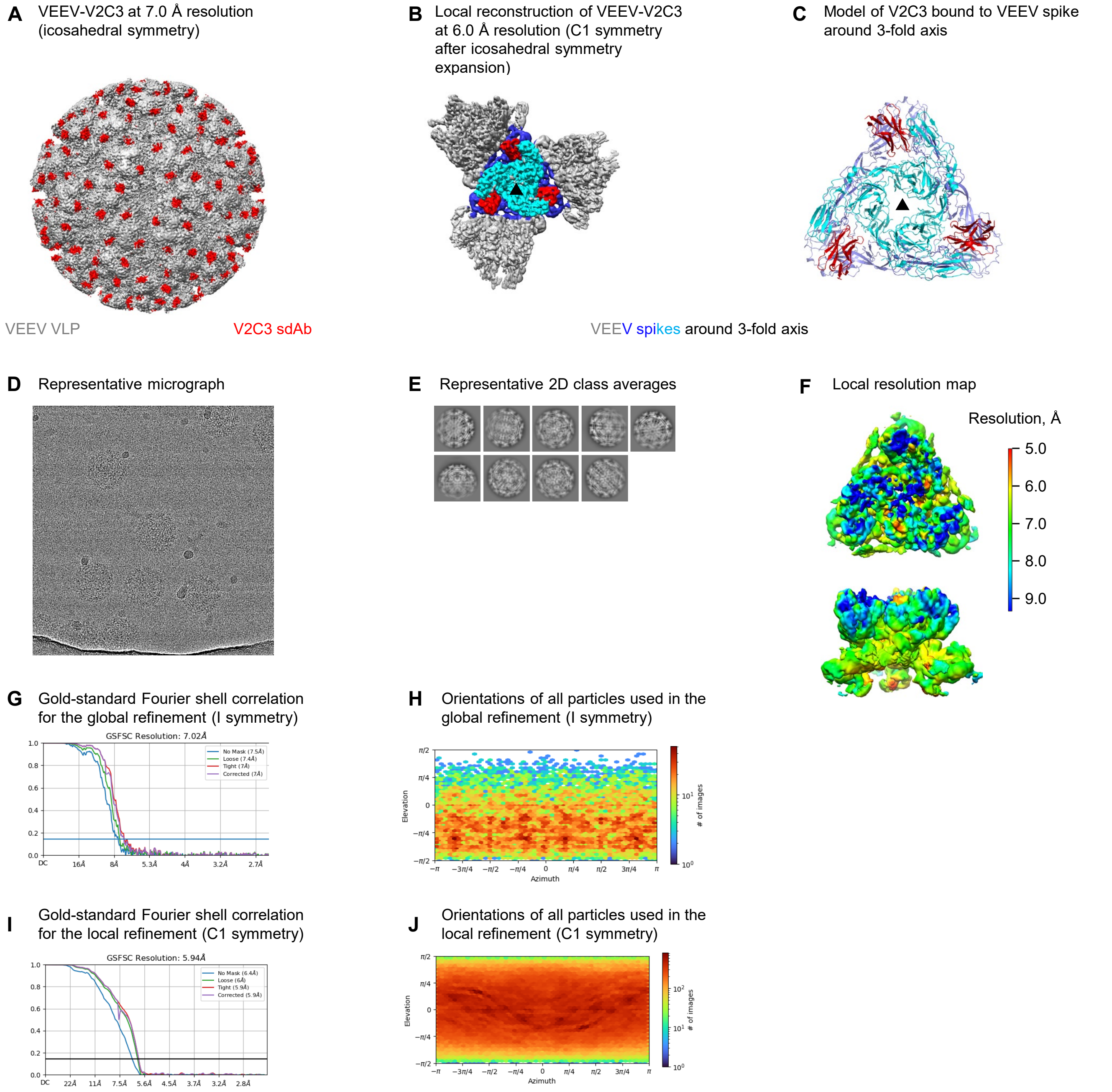

Fig S6 Cryo-EM structure of V2C3 sdAb in complex with VEEV VLP. (**A**) Cryo-EM reconstruction of VEEV-V2C3 complex at 7.0 Å resolution. V2C3 is shown in red. (**B**) Local reconstruction of four VEEV trimers after symmetry expansion arranged around an icosahedral 3-fold axis, with E1 subunit in slate blue and E2 subunit in cyan for the central spike, and grey for the other peripheral three spikes and V2C3 in red. (**C**) Model of VEEV-V2C3 complex colored as panel **B.** (**D**) representative micrograph. (**E**) Representative 2D class averages. (**F**) Local resolution map. (**G**) Gold-standard Fourier shell correlation for the entire complex after global refinement. (**H**) Heatmap showing the orientations of all particles used in the global refinement. (**I**) Gold-standard Fourier shell correlation for the four spikes arranged around icosahedral 3-fold axis after symmetry expansion and local refinement. (**J**) Heatmap showing the orientations of all particles used in local refinement.

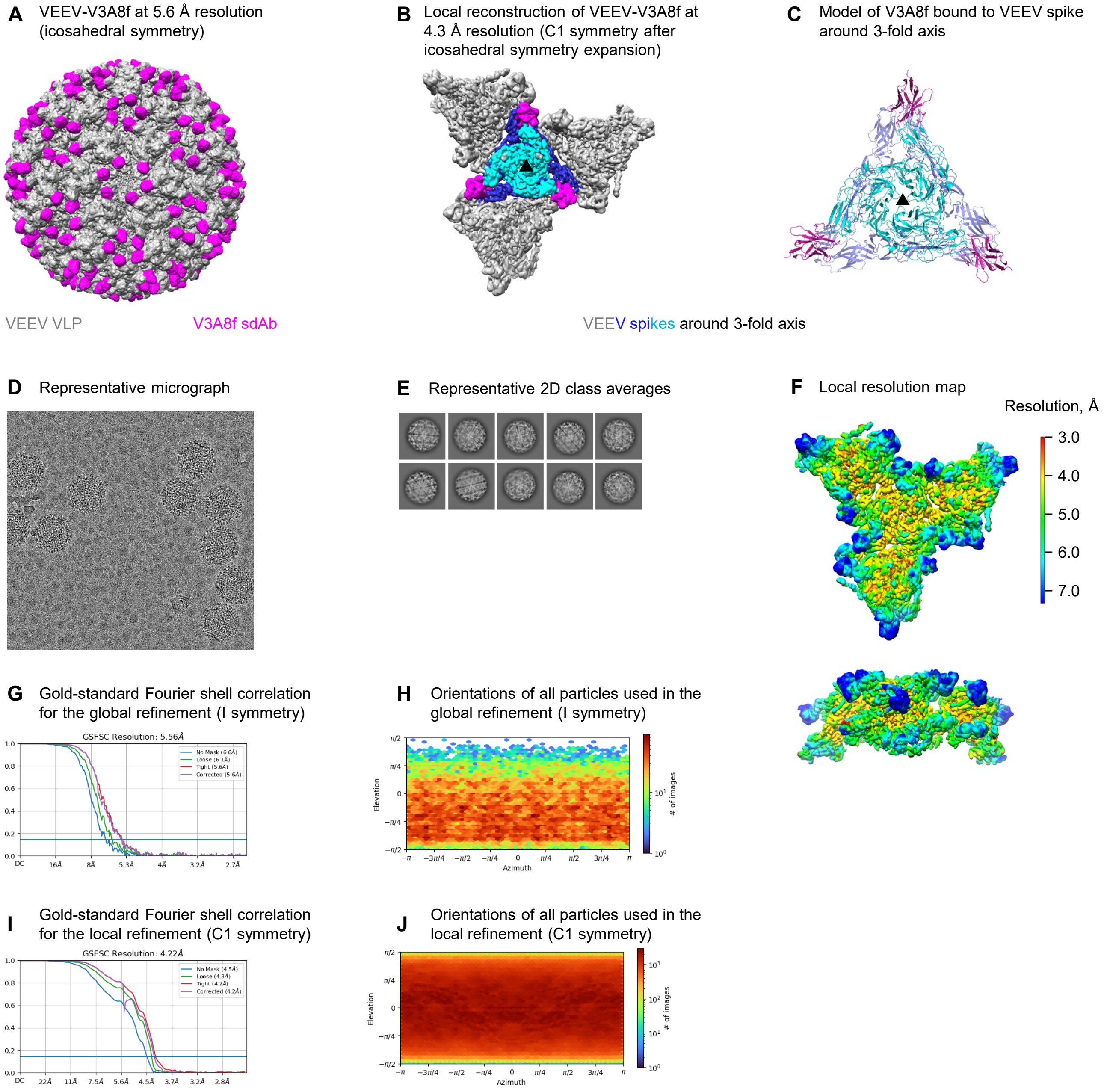

Fig S7 Cryo-EM structure of V3A8f sdAb in complex with VEEV VLP. (**A**) Cryo-EM reconstruction of VEEV-V3A8f complex at 5.6 Å resolution. V3A8f is shown in magenta. (**B**) Local reconstruction of four VEEV trimers after symmetry expansion arranged around an icosahedral 3-fold axis, with E1 subunit in slate blue and E2 subunit in cyan for the central spike, and grey for the other peripheral three spikes and V3A8f in magenta. (**C**) Model of VEEV-V3A8f complex colored as panel **B.** (**D**) representative micrograph. (**E**) Representative 2D class averages. (**F**) Local resolution map. (**G**) Gold-standard Fourier shell correlation for the entire complex after global refinement. (**H**) Heatmap showing the orientations of all particles used in the global refinement. (**I**) Gold-standard Fourier shell correlation for the four spikes arranged around icosahedral 3-fold axis after symmetry expansion and local refinement. (**J**) Heatmap showing the orientations of all particles used in local refinement.

Table S3 Cryo-EM data collection, refinement, and validation statistics for VEEV VLP-sdAb complexes

	VEEV-V2B3 (EMD-49740) (PDB 9NRX)	VEEV-V2C3 (EMD-49741) (PDB 9NRY)	VEEV-V3A8f (EMD-49742) (PDB 9NRZ)
**Data collection and processing**			
Magnification	81,000	81,000	81,000
Voltage (kV)	300	300	300
Electron exposure (e–/Å^2)	40.0	40.0	40.0
Defocus range (μm)	-0.8 to -2.0	-0.8 to -2.0	-0.8 to -2.0
Pixel size (Å)	1.11 / 1.246	1.11 / 1.246	1.11 / 1.246
Symmetry imposed	I / C1	I / C1	I / C1
Initial particle images (no.)	41,404 / I	31,101 / I	66,222 / I
Final particle images (no.)	2,451,747 / C1	726,375 / C1	3,972,522 / C1
Map resolution (Å)	4.6	6.0	4.3
FSC threshold	0.143	0.143	0.143
**Refinement**			
Initial model used (PDB code)	7FFE	7FFE	7FFE
Model resolution (Å)	4.6	6.0	4.3
FSC threshold	0.143	0.143	0.143
Model composition			
Non-hydrogen atoms	71,157	19,692	19,971
Protein residues	9,198	2,547	2,583
Ligands			
B factors (Å^2)			
Protein	148.2	198.7	106.7
Ligand			
R.m.s. deviations			
Bond lengths (Å)	0.004	0.004	0.004
Bond angles (°)	0.74	0.74	0.74
Validation			
MolProbity score	1.6	1.7	1.7
Clashscore	4.2	6.2	4.9
Poor rotamers (%)	0.0	0.0	0.0
Ramachandran plot			
Favored (%)	94.2	94.1	94.2
Allowed (%)	5.8	5.9	5.8
Disallowed (%)	0.0	0.0	0.0

Table S4 VEEV VLP-V2B3 sdAb interface details

<table>
<tr><th>Residue</th><th>Bond</th><th>Epitope BSA, Å²</th><th>Residue</th><th>Bond</th><th>Paratope BSA, Å²</th></tr>
<tr><td>VEEV E1 trimer 1</td><td></td><td></td><td>V2B3</td><td></td><td></td></tr>
<tr><td>B:ILE 39</td><td></td><td>16 |||</td><td>Z:SER 30</td><td></td><td>12 |||</td></tr>
<tr><td>B:PRO 40</td><td></td><td>2 ||</td><td>Z:ASN 32</td><td>H</td><td>87 |||||||||</td></tr>
<tr><td>B:THR 41</td><td></td><td>56 ||||||||</td><td>Z:VAL 33</td><td></td><td>17 ||||||</td></tr>
<tr><td>B:VAL 42</td><td>H</td><td>51 |||||||||</td><td>Z:ARG 45</td><td></td><td>16 ||</td></tr>
<tr><td>B:ASN 43</td><td></td><td>45 ||||||</td><td>Z:LYS 50</td><td></td><td>25 ||||</td></tr>
<tr><td>B:LEU 44</td><td></td><td>81 ||||||||||</td><td>Z:SER 52</td><td></td><td>28 |||||||||</td></tr>
<tr><td>B:VAL 47</td><td></td><td>7 |||||</td><td>Z:GLY 54</td><td></td><td>16 ||||</td></tr>
<tr><td>B:ASP 112</td><td></td><td>36 |||</td><td>Z:PRO 55</td><td></td><td>16 ||</td></tr>
<tr><td>B:CYS 114</td><td></td><td>4 |||</td><td>Z:PHE 56</td><td></td><td>89 ||||||</td></tr>
<tr><td>B:LEU 115</td><td></td><td>134 ||||||||||</td><td>Z:LEU 58</td><td></td><td>28 |||</td></tr>
<tr><td>B:ALA 116</td><td></td><td>8 |</td><td>Z:GLU 95</td><td></td><td>26 ||||</td></tr>
<tr><td>B:HIS 118</td><td></td><td>16 ||||</td><td>Z:GLY 96</td><td></td><td>34 |||||||||</td></tr>
<tr><td>B:GLU 120</td><td>H</td><td>18 |||||||</td><td>Z:LEU 97</td><td></td><td>15 |||||</td></tr>
<tr><td>B:TYR 122</td><td></td><td>19 |||||||</td><td>Z:ARG 98</td><td></td><td>93 ||||||</td></tr>
<tr><td>B:LYS 123</td><td></td><td>28 ||||</td><td>Z:TYR 99</td><td></td><td>7 |||</td></tr>
<tr><td>B:HIS 125</td><td>H</td><td>22 ||</td><td>Z:TYR 100D</td><td>H</td><td>134 |||||||</td></tr>
<tr><td>B:THR 126</td><td></td><td>8 |</td><td>Z:GLY 100E</td><td>H</td><td>34 ||||||||</td></tr>
<tr><td>B:GLN 179</td><td></td><td>7 ||</td><td>Z:PRO 100F</td><td></td><td>21 |||</td></tr>
<tr><td>B:ARG 206</td><td></td><td>26 |||</td><td>Z:SER 100G</td><td>H</td><td>71 |||||||||</td></tr>
<tr><td>B:THR 207</td><td></td><td>31 ||||||</td><td>Z:ALA 101</td><td></td><td>30 ||||||||</td></tr>
<tr><td>B:VAL 208</td><td></td><td>64 ||||||||</td><td>Z:TRP 103</td><td>H</td><td>31 ||||</td></tr>
<tr><td>B:SER 209</td><td></td><td>32 ||||</td><td></td><td></td><td></td></tr>
<tr><td>B:THR 263</td><td></td><td>3 |</td><td></td><td></td><td></td></tr>
<tr><td>B:ASN 264</td><td>H</td><td>88 |||||||</td><td></td><td></td><td></td></tr>
<tr><td>B:PRO 265</td><td></td><td>43 ||||||</td><td></td><td></td><td></td></tr>
<tr><td>B:ILE 266</td><td>H</td><td>10 ||||||||</td><td></td><td></td><td></td></tr>
<tr><td>VEEV E2 trimer 1</td><td></td><td></td><td>V2B3</td><td></td><td></td></tr>
<tr><td>Q:ASP 157</td><td></td><td>25 ||||</td><td>Z:GLU 46</td><td></td><td>7 |</td></tr>
<tr><td>Q:GLN 159</td><td></td><td>59 |||||||</td><td>Z:LEU 47</td><td></td><td>21 |||</td></tr>
<tr><td>Q:ASN 160</td><td></td><td>8 ||</td><td>Z:LYS 50</td><td></td><td>5 |</td></tr>
<tr><td>Q:ARG 161</td><td></td><td>68 |||||||||</td><td>Z:PRO 55</td><td></td><td>27 |||</td></tr>
<tr><td>Q:GLY 162</td><td></td><td>62 ||||||||</td><td>Z:PHE 56</td><td></td><td>56 ||||</td></tr>
<tr><td>Q:ALA 163</td><td></td><td>12 ||||</td><td>Z:THR 57</td><td></td><td>33 |||||||</td></tr>
<tr><td>Q:LEU 260</td><td></td><td>13 |||</td><td>Z:LEU 58</td><td></td><td>68 |||||||</td></tr>
<tr><td>Q:LEU 261</td><td></td><td>14 ||</td><td>Z:TYR 59</td><td></td><td>64 |||||||</td></tr>
<tr><td>Q:ALA 262</td><td></td><td>26 |||||||</td><td>Z:ALA 60</td><td></td><td>4 ||</td></tr>
<tr><td>Q:ASP 263</td><td></td><td>35 ||||</td><td>Z:ASP 61</td><td></td><td>15 ||</td></tr>
<tr><td>Q:LYS 265</td><td></td><td>22 ||</td><td>Z:LYS 64</td><td></td><td>27 |||</td></tr>
<tr><td></td><td></td><td></td><td>Z:GLY 65</td><td></td><td>18 |||</td></tr>
<tr><td></td><td></td><td></td><td>Z:PHE 67</td><td></td><td>2 |||</td></tr>
<tr><td></td><td></td><td></td><td>Z:THR 68</td><td></td><td>3 |</td></tr>
<tr><td>VEEV E1 trimer 2</td><td></td><td></td><td>V2B3</td><td></td><td></td></tr>
<tr><td>v:ILE 39</td><td></td><td>41 ||||||</td><td>Z:GLU 1</td><td></td><td>68 ||||</td></tr>
<tr><td>v:PRO 40</td><td>H</td><td>3 |||</td><td>Z:GLN 3</td><td></td><td>16 ||</td></tr>
<tr><td>v:THR 41</td><td></td><td>46 ||||||||</td><td>Z:GLN 5</td><td></td><td>19 |||</td></tr>
<tr><td>v:VAL 42</td><td>H</td><td>24 |||||</td><td>Z:SER 100A</td><td></td><td>46 |||||</td></tr>
<tr><td>v:ASN 43</td><td></td><td>7 |</td><td>Z:GLY 100B</td><td></td><td>2 |</td></tr>
<tr><td>v:LEU 44</td><td></td><td>40 |||||</td><td>Z:THR 100C</td><td>H</td><td>90 ||||||||</td></tr>
<tr><td>v:HIS 125</td><td></td><td>5 |</td><td>Z:TYR 100D</td><td></td><td>41 ||</td></tr>
<tr><td>v:THR 126</td><td></td><td>14 ||</td><td></td><td></td><td></td></tr>
<tr><td>v:ARG 206</td><td></td><td>3 |</td><td></td><td></td><td></td></tr>
<tr><td>v:THR 207</td><td></td><td>33 ||||||</td><td></td><td></td><td></td></tr>
<tr><td>v:SER 209</td><td></td><td>19 ||</td><td></td><td></td><td></td></tr>
<tr><td>v:PRO 265</td><td></td><td>4 |</td><td></td><td></td><td></td></tr>
</table>

https://www.ebi.ac.uk/pdbe/pisa/

BSA Buried Surface Area, Å²
HSDC Residues making **H**ydrogen/**D**isulphide bond, **S**alt bridge or **C**ovalent link
|||| Buried area percentage, one bar per 10%

Table S5 VEEV VLP-V2C3 sdAb interface details

<table>
<tr><th>Residue</th><th>Bond</th><th>Epitope BSA, Å²</th><th>Residue</th><th>Bond</th><th>Paratope BSA, Å²</th></tr>
<tr><td>VEEV E1</td><td></td><td></td><td>V2C3</td><td></td><td></td></tr>
<tr><td>B:THR 82</td><td></td><td>2 |</td><td>X:GLU 30</td><td>H</td><td>33 ||||</td></tr>
<tr><td>B:GLY 83</td><td></td><td>34 ||||||||</td><td>X:TYR 31</td><td>H</td><td>116 |||||||</td></tr>
<tr><td>B:VAL 84</td><td></td><td>4 |||</td><td>X:TYR 32</td><td></td><td>4 ||</td></tr>
<tr><td>B:TYR 85</td><td>H</td><td>76 |||||||||</td><td>X:THR 33</td><td></td><td>19 ||||||||</td></tr>
<tr><td>B:PHE 87</td><td></td><td>77 |||||||||</td><td>X:TYR 37</td><td></td><td>11 ||||</td></tr>
<tr><td>B:MET 88</td><td></td><td>6 |</td><td>X:LEU 50</td><td></td><td>27 |||||||</td></tr>
<tr><td>B:ALA 92</td><td></td><td>7 ||</td><td>X:ASN 52</td><td></td><td>59 ||||||||||</td></tr>
<tr><td>B:ASP 97</td><td></td><td>45 |||||</td><td>X:ASN 54</td><td>H</td><td>38 ||||</td></tr>
<tr><td>B:THR 98</td><td></td><td>28 ||||</td><td>X:GLY 55</td><td></td><td>47 ||||||</td></tr>
<tr><td>B:GLN 222</td><td>H</td><td>18 ||</td><td>X:GLY 56</td><td></td><td>13 ||||</td></tr>
<tr><td>B:ARG 223</td><td>H</td><td>54 |||||</td><td>X:SER 57</td><td></td><td>7 ||</td></tr>
<tr><td>B:PRO 224</td><td></td><td>5 |||</td><td>X:ASN 58</td><td>H</td><td>30 |||||</td></tr>
<tr><td>B:LYS 225</td><td>H</td><td>75 ||||||</td><td>X:ARG 93</td><td></td><td>3 |</td></tr>
<tr><td>B:ALA 226</td><td></td><td>63 ||||||||||</td><td>X:PHE 95</td><td></td><td>84 |||||||||</td></tr>
<tr><td>B:GLY 227</td><td></td><td>32 |||||||||</td><td>X:GLY 96</td><td></td><td>21 |||||||</td></tr>
<tr><td></td><td></td><td></td><td>X:PRO 97</td><td></td><td>43 ||||</td></tr>
<tr><td></td><td></td><td></td><td>X:ASP 101</td><td></td><td>5 |</td></tr>
<tr><td>VEEV E2</td><td></td><td></td><td>V2C3</td><td></td><td></td></tr>
<tr><td>Q:ARG 18</td><td></td><td>21 |||</td><td>X:ARG 47</td><td>S</td><td>52 ||||||</td></tr>
<tr><td>Q:SER 26</td><td></td><td>7 ||</td><td>X:ASN 54</td><td></td><td>25 |||</td></tr>
<tr><td>Q:HIS 28</td><td></td><td>12 |</td><td>X:GLY 55</td><td></td><td>16 ||</td></tr>
<tr><td>Q:LEU 181</td><td></td><td>5 |</td><td>X:ASN 58</td><td></td><td>22 ||||</td></tr>
<tr><td>Q:LYS 222</td><td>H</td><td>80 ||||||</td><td>X:VAL 59</td><td></td><td>4 |</td></tr>
<tr><td>Q:LYS 223</td><td>S</td><td>37 ||||</td><td>X:ALA 60</td><td></td><td>6 ||||||||||</td></tr>
<tr><td>Q:GLU 224</td><td>S</td><td>59 ||||||</td><td>X:GLY 61</td><td></td><td>29 |||||</td></tr>
<tr><td>Q:GLN 225</td><td></td><td>3 |</td><td>X:SER 62</td><td>H</td><td>28 |||</td></tr>
<tr><td>Q:ARG 227</td><td></td><td>11 ||</td><td>X:GLU 64</td><td>S</td><td>44 ||||</td></tr>
</table>

https://www.ebi.ac.uk/pdbe/pisa/

BSA Buried Surface Area, Å²
HSDC Residues making **H**ydrogen/**D**isulphide bond, **S**alt bridge or **C**ovalent link
|||| Buried area percentage, one bar per 10%

Table S6 VEEV VLP-V3A8f sdAb interface details

<table>
<tr><th>Residue</th><th>Bond</th><th>Epitope BSA, Å²</th><th>Residue</th><th>Bond</th><th>Paratope BSA, Å²</th></tr>
<tr><td>VEEV E1</td><td></td><td></td><td>V3A8f</td><td></td><td></td></tr>
<tr><td>B:LYS 61</td><td></td><td>9 |</td><td>X:ARG 53</td><td></td><td>16 |</td></tr>
<tr><td>B:CYS 62</td><td></td><td>5 ||</td><td>X:ASP 54</td><td></td><td>14 ||</td></tr>
<tr><td>B:CYS 63</td><td></td><td>31 |||||||</td><td>X:MET 97</td><td></td><td>21 ||||</td></tr>
<tr><td>B:CYS 94</td><td></td><td>3 |</td><td>X:ALA 98</td><td></td><td>11 ||</td></tr>
<tr><td>B:PHE 95</td><td></td><td>14 |</td><td>X:GLN 99</td><td></td><td>57 ||||</td></tr>
<tr><td>B:CYS 96</td><td></td><td>14 ||||</td><td></td><td></td><td></td></tr>
<tr><td>B:ASP 97</td><td></td><td>8 |</td><td></td><td></td><td></td></tr>
<tr><td>B:THR 98</td><td></td><td>7 |</td><td></td><td></td><td></td></tr>
<tr><td>B:GLU 99</td><td></td><td>24 |||</td><td></td><td></td><td></td></tr>
<tr><td>VEEV E2</td><td></td><td></td><td>V3A8f</td><td></td><td></td></tr>
<tr><td>Q:GLU 199</td><td></td><td>7 ||</td><td>X:SER 50</td><td></td><td>6 |||||</td></tr>
<tr><td>Q:GLU 201</td><td>H</td><td>31 ||||</td><td>X:ASP 54</td><td></td><td>29 |||</td></tr>
<tr><td>Q:CYS 202</td><td></td><td>29 ||||||||</td><td>X:SER 56</td><td></td><td>26 ||||||</td></tr>
<tr><td>Q:GLY 203</td><td></td><td>39 ||||||||</td><td>X:THR 57</td><td></td><td>10 ||</td></tr>
<tr><td>Q:GLY 204</td><td></td><td>45 |||||||||</td><td>X:TYR 58</td><td></td><td>97 |||||||</td></tr>
<tr><td>Q:THR 205</td><td>H</td><td>81 ||||||||</td><td>X:TYR 59</td><td>H</td><td>21 ||||</td></tr>
<tr><td>Q:LYS 206</td><td></td><td>18 |||</td><td>X:LEU 64</td><td></td><td>11 |</td></tr>
<tr><td>Q:GLN 219</td><td>H</td><td>92 ||||||</td><td>X:GLN 99</td><td></td><td>44 |||</td></tr>
<tr><td>Q:GLN 225</td><td></td><td>16 ||</td><td>X:THR 100A</td><td>H</td><td>51 ||||||||</td></tr>
<tr><td></td><td></td><td></td><td>X:GLN 100B</td><td></td><td>26 |||</td></tr>
<tr><td></td><td></td><td></td><td>X:GLY 100C</td><td>H</td><td>7 ||</td></tr>
</table>

https://www.ebi.ac.uk/pdbe/pisa/

BSA Buried Surface Area, Å²
HSDC Residues making **H**ydrogen/**D**isulphide bond, **S**alt bridge or **C**ovalent link
|||| Buried area percentage, one bar per 10%
